# Impact of AmpC Derepression on Fitness and Virulence: the Mechanism or the Pathway?

**DOI:** 10.1128/mBio.01783-16

**Published:** 2016-10-25

**Authors:** Marcelo Pérez-Gallego, Gabriel Torrens, Jane Castillo-Vera, Bartolomé Moya, Laura Zamorano, Gabriel Cabot, Kjell Hultenby, Sebastián Albertí, Peter Mellroth, Birgitta Henriques-Normark, Staffan Normark, Antonio Oliver, Carlos Juan

**Affiliations:** a Servicio de Microbiología and Unidad de Investigación, Hospital Son Espases, Instituto de Investigación Sanitaria de Palma (IdISPa), Palma de Mallorca, Spain; b Department of Laboratory Medicine, Karolinska Institutet and University Hospital, Huddinge, Sweden; c Instituto Universitario de Investigación en Ciencias de la Salud, Universidad de las Islas Baleares, Palma de Mallorca, Spain; d Department of Microbiology, Tumor and Cell Biology, Karolinska Institutet, Stockholm, Sweden; e Department of Clinical Microbiology, Karolinska University Hospital, Stockholm, Sweden

## Abstract

Understanding the interplay between antibiotic resistance and bacterial fitness and virulence is essential to guide individual treatments and improve global antibiotic policies. A paradigmatic example of a resistance mechanism is the intrinsic inducible chromosomal β-lactamase AmpC from multiple Gram-negative bacteria, including *Pseudomonas aeruginosa*, a major nosocomial pathogen. The regulation of *ampC* expression is intimately linked to peptidoglycan recycling, and AmpC-mediated β-lactam resistance is frequently mediated by inactivating mutations in *ampD*, encoding an *N*-acetyl-anhydromuramyl-l-alanine amidase, affecting the levels of *ampC*-activating muropeptides. Here we dissect the impact of the multiple pathways causing AmpC hyperproduction on *P. aeruginosa* fitness and virulence. Through a detailed analysis, we demonstrate that the lack of all three *P. aeruginosa* AmpD amidases causes a dramatic effect in fitness and pathogenicity, severely compromising growth rates, motility, and cytotoxicity; the latter effect is likely achieved by repressing key virulence factors, such as protease LasA, phospholipase C, or type III secretion system components. We also show that *ampC* overexpression is required but not sufficient to confer the growth-motility-cytotoxicity impaired phenotype and that alternative pathways leading to similar levels of *ampC* hyperexpression and resistance, such as those involving PBP4, had no fitness-virulence cost. Further analysis indicated that fitness-virulence impairment is caused by overexpressing *ampC* in the absence of cell wall recycling, as reproduced by expressing *ampC* from a plasmid in an AmpG (muropeptide permease)-deficient background. Thus, our findings represent a major step in the understanding of β-lactam resistance biology and its interplay with fitness and pathogenesis.

## IMPORTANCE

Understanding the impact of antibiotic resistance mechanisms on bacterial pathogenesis is critical to curb the spread of antibiotic resistance. A particularly noteworthy antibiotic resistance mechanism is the β-lactamase AmpC, produced by *Pseudomonas aeruginosa*, a major pathogen causing hospital-acquired infections. The regulation of AmpC is linked to the cell wall recycling pathways, and frequently, resistance to β-lactams is caused by mutation of several of the components of the cell wall recycling pathways such as AmpD. Here we dissect the impact of the pathways for AmpC hyperproduction on virulence, showing that the lack of all three *P. aeruginosa* AmpD amidases causes a major effect in fitness and pathogenicity, compromising growth, motility, and cytotoxicity. Further analysis indicated that fitness-virulence impairment is specifically caused by the hyperproduction of AmpC in the absence of cell wall recycling. Our work provides valuable information for delineating future strategies for combating *P. aeruginosa* infections by simultaneously targeting virulence and antibiotic resistance.

## INTRODUCTION

Continuously increasing antimicrobial resistance, added to the limited development of new antibiotics, is of growing concern since it severely compromises our therapeutic arsenal to fight life-threatening bacterial infections (1). Understanding the interplay between antibiotic resistance and bacterial fitness and virulence is thus of paramount relevance for guiding individual treatments and global antibiotic policies. While there is a well-established body of evidence indicating that generally antibiotic resistance is associated with a biological cost, an enormous variability of the cost has been reported, depending on the antibiotic, pathogen, or genetic context (2, 3). Moreover, in many instances, the precise factors driving the fitness effects remain elusive. Likewise, reduced fitness is expected to directly impair bacterial virulence, but the interplay between virulence and antibiotic resistance is far more complex, including regulatory circuits controlling both traits (4, 5).

Resistance by modification of an antibiotic target such as DNA gyrase (quinolones) or the β subunit of RNA polymerase (rifampin) is frequently associated with a direct biological cost, but the basis underlying the fitness and virulence effects associated with the resistance due to detoxifying mechanisms such as efflux pumps, or antibiotic-inactivating enzymes (like β-lactamases) is far more complex and controversial (2, 6). Potential explanations range from the simple energetic cost of expressing the resistance mechanisms to direct mechanistic side effects (such as extrusion of compounds relevant for cell physiology or virulence by efflux pumps or the modification of cell components by antibiotic-inactivating enzymes) or indirect effects produced by the specific mutations (generally in regulatory genes) leading to the expression of the resistance mechanisms.

A complex example is the intrinsic inducible chromosomal β-lactamase AmpC from multiple Gram-negative rods, including most enterobacteria and *Pseudomonas aeruginosa*, a major nosocomial pathogen considered a paradigmatic model of antimicrobial resistance and virulence (7, 8). Mutational overexpression of *ampC* is a very frequent cause of resistance to the antipseudomonal penicillins and cephalosporins (9, 10).

Multiple genes are involved in the regulation of *ampC* expression, a process that is intimately linked to peptidoglycan recycling (11, 12). The *ampG* gene encodes an inner membrane permease for GlcNAc-1,6-anhydromuropeptides, which are peptidoglycan catabolites that, upon entry into the cytosol, are processed by the β-*N*-acetylglucosaminidase NagZ to generate 1,6-anhydromuropeptides (13, 14). The 1,6-anhydromuropeptide products of NagZ are thought to induce AmpC production by interacting with the LysR-type transcriptional regulator AmpR (15–17). During bacterial growth, 1,6-anhydromuropeptides are processed by the *N*-acetyl-anhydromuramyl-l-alanine amidase AmpD, avoiding *ampC* induction (18). During growth in the presence of strong β-lactamase inducers (such as carbapenems), large amounts of muropeptides are generated and accumulate in the cytoplasm which leads to AmpR-mediated induction of *ampC* expression (16, 17). Mutational inactivation of AmpD also leads to the accumulation of 1,6-anhydromuropeptides and to *ampC* hyperexpression, even in the absence of β-lactamase inducers, resulting in a constitutively derepressed resistance phenotype due to AmpC overproduction (9, 19). Further studies showed that *P. aeruginosa* has, in addition to the cytoplasmic AmpD, two periplasmic *N*-acetyl-anhydromuramyl-l-alanine amidases (AmpDh2 and AmpDh3) and that their sequential inactivation leads to a stepwise upregulation of *ampC*, reaching full derepression with very high levels (>1,000-fold with respect to the wild-type level) of *ampC* expression and clinical β-lactam resistance in the triple mutant (20). This full *ampC* derepression through inactivation of the three amidases has been associated with fitness and virulence impairment (21).

More-recent work showed that one-step clinical resistance in *P. aeruginosa* frequently results from the inactivation of *dacB* encoding the nonessential penicillin-binding protein 4 (PBP4) (22). The dd-carboxypeptidase and endopeptidase PBP4 has been shown to play a sentinel role for cell wall damage by β-lactams, producing a complex resistance response, triggering overproduction of the chromosomal β-lactamase AmpC and specific activation of the CreBC (BlrAB) two-component regulator (23–26). Moreover, the *ampC* transcriptional regulator AmpR has been shown to have global regulatory functions, including several virulence genes (27). Illustrative schemes of the complex *ampC* regulation pathways have been published previously (12, 22).

Altogether, the mentioned studies suggest that an interplay between *ampC* regulation, β-lactam resistance, cell wall recycling, fitness, and virulence might exist. However, the available information is disperse and limited, failing to clarify key aspects such as whether overexpression of *ampC* (the mechanism) or the blocking of peptidoglycan recycling (the pathway) is impacting fitness and virulence or whether it is restricted to an energy burden matter or, alternatively, to specific virulence responses. Using the two reference *P. aeruginosa* strains PAO1 and PA14, we demonstrate that the lack of all three AmpD amidases has a major effect on fitness and pathogenicity, severely compromising growth rates, motility, and cytotoxicity, the latter being likely due to repression of key virulence factors. Moreover, we show that *ampC* overexpression is required but not sufficient to confer the growth-motility-cytotoxicity impaired phenotype (GMC phenotype) and that alternative pathways such as those involving PBP4, leading to similar levels of *ampC* hyperexpression and β-lactam resistance avoid any defect in fitness or virulence. Finally, we demonstrate that the fitness-virulence impairment is due to overexpression of *ampC* in the absence of cell wall recycling, as reproduced when expressing *ampC* from a plasmid in an AmpG (muropeptide permease)-deficient background. Thus, our results elucidate a major pathway to β-lactam resistance side effects to the bacterial host and how they could contribute to the development of strategies for combating *P. aeruginosa* infections.

## RESULTS

### Overview of *ampC* derepression pathways.

Table 1 shows a comparative analysis of the effects on β-lactam resistance and *ampC* expression produced by single or multiple mutations in the genes *ampD*, *ampDh2*, and *ampDh3* for the three peptidoglycan-recycling amidases and/or *dacB* for PBP4 in *P*. *aeruginosa* reference strains PAO1 and PA14. Results for strain PAO1, in agreement with previous data (20, 22, 28), confirmed a stepwise increase of β-lactam MICs and *ampC* expression by inactivation of the three amidases, reaching high-level resistance and *ampC* values approximately 1,000-fold above the wild-type values in the triple mutant. The *ampC* expression levels and β-lactam MICs were slightly higher for the *ampD dacB* double mutant (Table 1). Overall, the results for strain PA14 followed the same trend with some differences. Basal expression of *ampC* in PA14 was slightly higher than in PAO1 but was much less responsive to mutation in *ampD* or *dacB*. Double *ampD ampDh3* inactivation in PA14 produced a major effect on *ampC* expression (approximately 1,000-fold increase), which was not much further increased in the *ampD* triple mutant. The impact on β-lactam resistance and *ampC* expression of the *ampD dacB* double mutant was lower than that of PAO1. In summary, similar >1,000-fold increases in *ampC* expression levels were obtained in the *ampD ampDh2 ampDh3* triple mutant and *ampD dacB* double mutant of PAO1 and in the *ampD ampDh2 ampDh3* triple mutant and *ampD ampDh3* double mutant of PA14 (Table 1).

**TABLE 1:** MICs of different antibiotics and *ampC* expression levels for strains PAO1 and PA14 and mutants derived from these two strains

Strain	MIC (µg/ml)^a^								Avg level of *ampC* expression ± SD^b^
	CAZ	FEP	PIP-TZ	ATM	IMP	MER	VAN	COL	
PAO1	1	1	4	1	1.5	0.38	512	0.75	1
PAΔD	6	4	64	6	1.5	1	512	0.75	63.3 ± 7.1
PAΔDDh2	16	12	96	16	1.5	1	512	0.75	79.5 ± 12.2
PAΔDDh3	48	32	>256	16	1.5	2	512	0.75	251.2 ± 51.9
PAΔDDh2Dh3	48	32	>256	32	1.5	2	512	0.75	1,225 ± 101
PAΔDDh2Dh3 + pUCPAD	1.5	1.5	6	1.5	1	0.5	512	0.75	2.9 ± 0.7
PAΔdacB	32	12	96	16	1.5	0.5	512	0.75	53 ± 22.2
PAΔdacBΔD	128	64	>256	48	2	2	512	0.75	1,770 ± 401
PAΔAG	1	1	3	1	0.38	0.38	512	0.75	−1.1 ± 0.4
PAO1 + pUCPAC	24	16	96	16	1.5	1.5	512	0.75	1,925.5 ± 514
PAΔAG + pUCPAC	24	16	96	16	1.5	1.5	512	0.75	1,480 ± 359.1
PA14	0.75	0.5	3	1	1	0.125	512	0.25	5.2 ± 2.9
PA14ΔD	1.5	1.5	4	3	1	0.19	512	0.25	8.9 ± 1.5
PA14ΔDh2	0.75	0.5	3	1	1	0.125	512	0.25	5.1 ± 1.2
PA14ΔDh3	0.75	0.5	3	1	1	0.125	512	0.25	4.5 ± 2.2
PA14ΔDh2Dh3	1	0.5	3	1	1	0.19	512	0.25	4.7 ± 1.5
PA14ΔDDh2	1	0.75	4	1.5	1	0.125	512	0.25	12 ± 2.1
PA14ΔDDh3	16	8	128	16	1	1.5	512	0.25	4,561 ± 570
PA14ΔDDh2Dh3	16	8	>256	24	1.5	1.5	512	0.25	5,569 ± 928
PA14ΔDDh2Dh3 + pUCPAD	2	1.5	6	3	1	0.25	512	0.25	152.2 ± 77.1
PA14ΔdacB	4	3	8	4	1	0.19	512	0.25	36 ± 10.2
PA14ΔdacBΔD	8	6	96	12	1	0.25	512	0.25	114.1 ± 42.1

a Abbreviations: CAZ, ceftazidime; FEP, cefepime; PIP-TZ, piperacillin-tazobactam; ATM, aztreonam; IMP, imipenem; MER, meropenem; VAN, vancomycin; COL, colistin.

b Relative amount of *ampC* mRNA compared to the amount expressed in strain PAO1.

### Impact of AmpC hyperproduction on growth rate and motility: not only a matter of metabolic cost.

**FIG 1:**
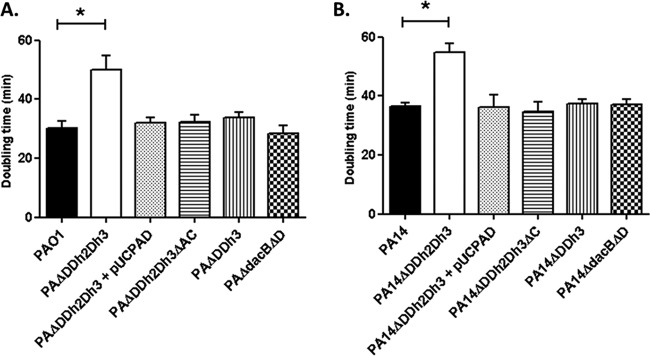
Duplication times of *P*. *aeruginosa* strains. Duplication times (in minutes) of exponentially growing cells of *P*. *aeruginosa* PAO1 (A) and PA14 (B) strains and derivatives in LB broth. Values are means plus standard deviations (SDs) (error bars) from three independent experiments. The values that are significantly different (*P* < 0.0001 by Student’s *t* test) from the value for the wild-type strain are shown by bars and an asterisk. The rest of the single and double *ampD* mutants of both PAO1 and PA14 strains showed wild-type doubling times (data not shown).

**FIG 2:**
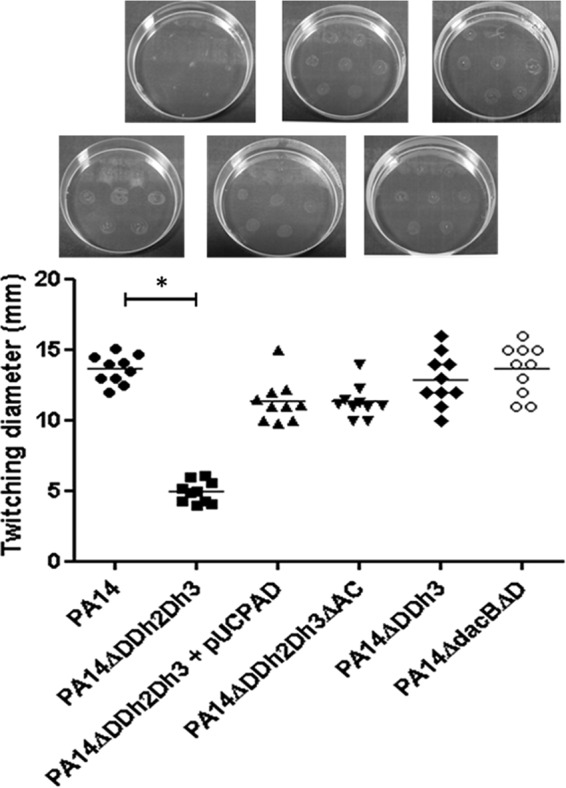
Twitching motility of strain PA14 and derived mutants. Colonies were inoculated as described in Materials and Methods. The diameters of the motility areas of 10 different colonies per strain were measured and plotted. Each symbol represents the value for one colony. Mean values for the 10 colonies are indicated by the short black bars for the strains. The values (means) that are significantly different (*P* < 0.0001 by Student’s *t* test) from the mean value for the wild-type strain are shown by a bar and asterisk. The rest of the single and double *ampD* mutants of strain PA14 showed wild-type twitching motility levels (not shown). The pictures above the graph show representative images of the corresponding strains.

**FIG 3:**
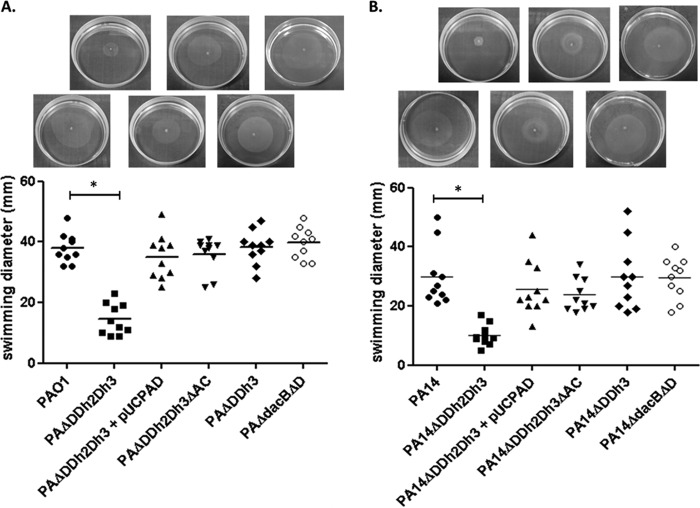
Swimming motility of strains PAO1 (A) and PA14 (B) and mutants derived from the two strains. Colonies were inoculated as described in Materials and Methods. The diameters of the motility areas of 10 different colonies per strain were measured. Each symbol represents the value for one colony, and the mean value for the 10 colonies is indicated by the short black bar. The values (means) that are significantly different (*P* < 0.0001 by Student’s *t* test) from the mean value for the wild-type strain are shown by a bar and asterisk. The rest of the single and double *ampD* mutants of both PAO1 and PA14 showed wild-type swimming motility levels (not shown). The pictures above the graph show representative images of the corresponding strains.

**FIG 4:**
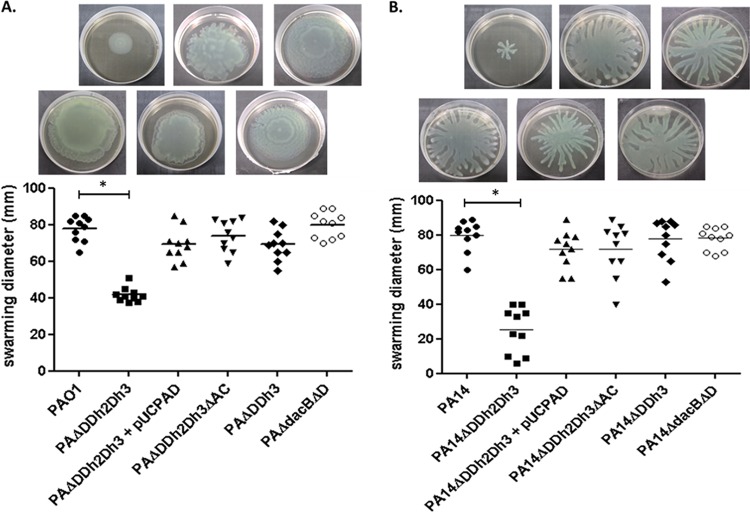
Swarming motility of strains PAO1 (A) and PA14 (B) and mutants derived from the two strains. Plates were inoculated as described in Materials and Methods. The diameters of the motility areas of 10 different colonies per strain were measured. Each symbol represents the value for one colony, and the mean value for the 10 colonies is indicated by the short black bar. The values (means) that are significantly different (*P* < 0.0001 by Student’s *t* test) from the mean value for the wild-type strain are shown by a bar and asterisk. The rest of the single and double *ampD* mutants of both PAO1 and PA14 showed wild-type swarming motility levels (not shown). The pictures above the graph show representative images of the corresponding strains.

In order to evaluate the potential fitness cost associated with AmpC hyperproduction and/or blocking of peptidoglycan recycling, the growth rates of the mutants discussed below were determined (Fig. 1). The simultaneous inactivation of the three amidases increased the doubling times of both PAO1 (49.9 min versus 30.1 min in the wild type) and PA14 (54.7 min versus 36.4 min in the wild type). Growth curves of the relevant strains are shown in Fig. S1 in the supplemental material. AmpC overexpression was required for the growth impairment, since knocking out *ampC* in the triple *ampD* mutant fully restored the wild-type growth rate for both PAO1 and PA14 strains. Likewise, *trans*-complementation with wild-type *ampD* also restored the wild-type growth rate. However, AmpC overexpression *per se* did not have a major impact on growth rates, since the *ampD dacB* and *ampD ampDh3* double mutants with similar *ampC* hyperexpression levels had the same generation times as strains PAO1 and PA14, respectively (Fig. 1). It can be deduced from these results that impaired peptidoglycan recycling and accumulation of muropeptides caused by the lack of amidase activity in the *ampD* triple mutant do not have a significant effect on growth rates. We next examined whether AmpC hyperproduction and/or blocking of peptidoglycan recycling affected bacterial motility. As for growth rates, inactivation of the three amidases had a substantial effect on the three types of motility, including twitching (Fig. 2), swimming (Fig. 3), and swarming (Fig. 4). The results were identical for PAO1 and PA14 derivatives; the only exception was that twitching could not be evaluated in PAO1 derivatives since the wild-type strain is defective in this type of motility. Again, *ampC* overexpression was required for the motility impairment, as knocking out *ampC* in the *ampD* triple mutant restored wild-type motility. Likewise, *trans*-complementation with wild-type *ampD* also restored the wild-type motility. However, *ampC* overexpression did not have a major effect on bacterial motility since the *ampC* hyperexpressing *ampD dacB* and *ampD ampDh3* double mutants had wild-type twitching, swimming, and swarming. Again, these results indicated that impaired peptidoglycan recycling and accumulation of muropeptides caused by lack of amidase activity in the *ampD* triple mutant do not have a significant effect on bacterial motility *per se*.

Thus, the very high-level *ampC* overexpression (>1,000-fold compared to the wild-type value) has a major impact on growth rate and motility, but only if it is associated with an amidase-deficient background. In other words, *ampC* overexpression is necessary but not sufficient for the observed growth- and motility-impaired phenotype, indicating a capital role of the pathway involved.

### Pathway-dependent effect of *ampC* derepression on virulence.

**FIG 5:**
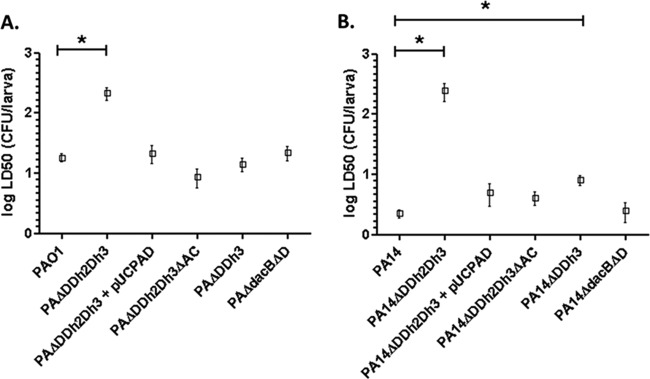
*Galleria mellonella* killing assays with strains derived from *P*. *aeruginosa* PAO1 (A) and PA14 (B). *G. mellonella* larvae were infected as described in Materials and Methods. LD_50_s were determined using a Probit model as previously described (53). Values are means (boxes) ± SD (error bars) from at least three independent experiments, using 10 larvae per strain in each experiment. To analyze the differences between the obtained LD_50_s, a Student’s *t* test was performed with the values of the three independently obtained LD_50_s. The values (means) that are significantly different (*P* < 0.005 by Student’s *t* test) from the mean value for the wild-type strain are shown by a bar and asterisk. The rest of the single and double *ampD* mutants of both PAO1 and PA14 strains showed wild-type virulence levels (data not shown).

We next analyzed whether AmpC hyperproduction and/or blocking of peptidoglycan recycling affects bacterial virulence in the *Galleria mellonella* killing assay (Fig. 5). The 50% lethal dose (LD_50_) of strain PA14 (ca. 2 CFU) is lower than that of strain PAO1 (ca. 18 CFU) due to the higher virulence of the former (29, 30). Inactivation of the three amidases produced a major increase (approximately 12-fold) of the LD_50_ of strain PAO1 (Fig. 5A), and the increase was much higher (approximately 110-fold) for the triple mutant of strain PA14. Interestingly, despite the major virulence difference of the wild-type strains, the PAO1 and PA14 triple amidase mutants had similar LD_50_s. As for growth rate and motility, wild-type virulence was restored upon inactivation of *ampC* and through *trans*-complementation with wild-type *ampD*. Thus, the effect was specific to the triple amidase mutants since the *ampD dacB* or *ampD ampDh3* mutants showed wild-type virulence, except for a very modest increase in the LD_50_ of the PA14 *ampD-ampDh3* mutant (Fig. 5B).

### *ampC* derepression does not cause major changes in cell envelope physiology.

To examine whether the effects on growth rates, motility, and virulence of the inactivation of the three *P. aeruginosa* amidases was a consequence of a modification of the cell envelope or related structures, wild-type PAO1 and its triple amidase mutant were analyzed by electron microscopy (see Fig. S2 in the supplemental material). Negative staining (Fig. S2A and B) showed no differences in the presence and width of flagella, whereas scanning electron microscopy (SEM) (Fig. S2C and D) did not reveal differences in cell shape or size. Moreover, the bacterial sections obtained for transmission electron microscopy (TEM) analysis did not show alterations in the morphology and width of the bacterial envelopes (Fig. S2E and F).

Additionally, we analyzed whether inactivation of the three amidases could modify the susceptibility to colistin, which targets the bacterial surface, or vancomycin, which targets the cell wall with an activity highly affected by the level of peptidoglycan cross-linking (23). Nevertheless, as shown in Table 1, colistin and vancomycin MICs were identical against mutant and wild-type strains. The only difference was that strain PAO1 (and derivatives) showed higher colistin MICs than strain PA14 did.

We next examined whether inactivation of the three amidases weakens the peptidoglycan, making the host more susceptible to killing by immune system components targeting the bacterial surface, such as the complement or neutrophils. To evaluate complement-mediating killing, strains PAO1 and PA14 and their respective triple amidase mutants were incubated with nonimmune human serum (NHS) and heat-inactivated nonimmune human serum (HI-NHS). Results shown in Fig. S3 in the supplemental material evidenced that strain PAO1 is more resistant to complement-mediated killing than strain PA14 is but revealed no differences between mutant and wild-type strains. To evaluate neutrophil killing, the percentages of bacterial survival after coincubation with freshly purified neutrophils was assessed, and the results are also shown in Fig. S3. However, no significant differences were found when comparing each wild-type strain with its corresponding triple amidase mutant, reaching in all cases a high rate of neutrophil-mediated bacterial death, close to 95%. Finally, when using HI-NHS instead of NHS, a general decrease in neutrophil bactericidal activity was observed as expected, given the almost complete reduction in opsonization capacity that heat inactivation causes in the complement cascade, but once more, no differences between wild-type and mutant strains were found.

### Cytotoxicity is specifically impaired by pathway-dependent *ampC* derepression.

We next analyzed whether the observed effect on virulence could also be caused by a decreased invasiveness or cytotoxicity mediated by *ampC* overexpression and/or blocking of peptidoglycan recycling. For this purpose, wild-type strains PAO1 (model invasive strain) and PA14 (model cytotoxic strain) and mutant derivatives were tested.

**FIG 6:**
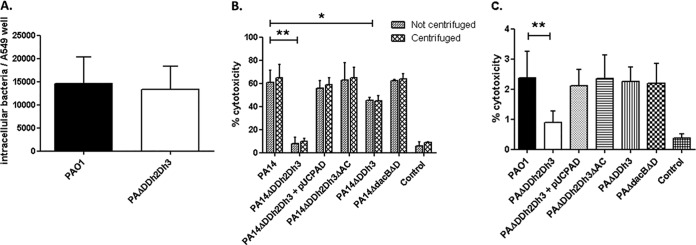
A549 cell invasion and cytotoxicity assays of strains PAO1 and PA14 and mutants derived from the two strains. (A) PAO1 invasion assay. The number of invasive CFU per well of A549 cells (multiplicity of infection [MOI] of 100) after 3 h of infection is shown. Values are means plus SD for at least three wells from three independent plates. (B and C) Cytotoxicity results after 3 h of infection (MOI of 100) of strains PA14 (B) and PAO1 (C). Values are means plus SD for at least three wells from three independent plates. The values that are significantly different by Student’s *t* test are indicated by bars and asterisks as follows: *, *P* < 0.05; **, *P* < 0.0001. The rest of the single and double *ampD* mutants of both PAO1 and PA14 showed wild-type levels of invasiveness and/or cytotoxicity (not shown).

To evaluate invasion capacity, A549 human type II alveolar epithelial cells were challenged with wild-type strain PAO1 or its triple amidase mutant, and the numbers of intracellular bacteria were assessed after 3 h of coincubation. However, as shown in Fig. 6A, no differences on intracellular bacteria numbers were found between strain PAO1 and the mutant. Invasion capacity could not be assessed for strain PA14, since fast cell lysis produced by this highly cytotoxic strain precluded the accurate quantification of intracellular bacteria.

On the other hand, cytotoxicity was evaluated in strain PA14 and derivatives through the quantification of lactate dehydrogenase (LDH) release in culture supernatants (Fig. 6B). Interestingly, cytotoxicity was nearly fully abolished in the triple amidase mutant. Moreover, once more, cytotoxicity was restored upon *ampC* inactivation or *trans*-complementation with wild-type *ampD*. Likewise, the effect was specific to the triple amidase mutant, since the *ampD dacB* mutant showed wild-type cytotoxicity and the *ampD ampDh3* mutant displayed only a slight reduction (Fig. 6B). To rule out the possibility that the abolishment of cytotoxicity could be a consequence of the mentioned reduced motility, the assays were repeated after adding a plate centrifugation step to ensure close contact between bacterial and epithelial cells, but as can be observed in Fig. 6B, the same results were obtained. Additionally, the LDH assays were also performed with PAO1 derivatives and despite the very low cytotoxicity of this strain, the same trend was evidenced (Fig. 6C).

Finally, to address whether the triple amidase inactivation could trigger effects changing the proinflammatory capacity of the peptidoglycan, we analyzed the interleukin 8 (IL-8) release in A549 cell cultures during coincubation with different stimuli. However, IL-8 release was not modified in the mutant strains compared to the wild-type strain (PAO1 or PA14) when using either intact cells (see Fig. S4A in the supplemental material), cell-free bacterial culture supernatants (Fig. S4B), or purified peptidoglycans (Fig. S4C). The only remarkable difference was that IL-8 release was higher when using PAO1 purified peptidoglycan compared to PA14 and the other way around for cell-free culture supernatants.

### Global transcriptomics reveals major specific effects of pathway-dependent *ampC* derepression on virulence gene expression.

We next analyzed whether the observed fitness and virulence effects could be consequences of modified gene expression. For this purpose, we performed a global transcriptomic analysis of the triple *ampD* mutant compared to strain PAO1 using the Affymetrix gene chips. Up to 81 genes showed a modified (≥2-fold) expression; 47 genes were upregulated, and 34 were downregulated (see Table S1 in the supplemental material). Upregulated genes obviously included *ampC*, but most other genes fell within the energy metabolism category and thus could be related to the lower growth rate of the *ampD* triple mutant. On the other hand, downregulated genes were significantly enriched in virulence genes. Indeed, downregulated genes included, among others, *lasA* (protease), *plcB* (phospholipase C), the *pilM-pilQ* operon (type IV pili), *wbpL* and *wbpH* (lipopolysaccharide [LPS] biosynthesis), and seven genes (*pcrV*, *pcrH*, *popB*, *popD*, *pscE*, *pscH*, and *pscI*) related to the type III secretion system (TTSS). Moreover, the level of expression of *exoS*, encoding a key cytotoxin secreted by the TTSS, was 1.9-fold lower in the triple *ampD* mutant than in wild-type PAO1.

**FIG 7:**
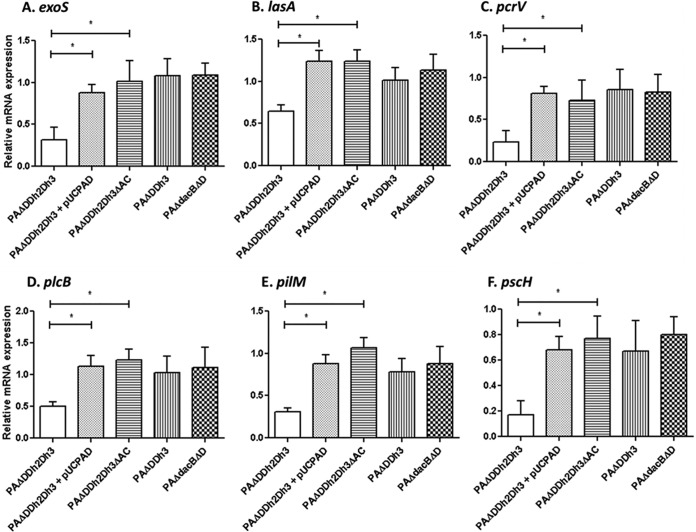
Expression of selected virulence genes in strains derived from *P*. *aeruginosa* PAO1, determined through RT-PCR following the protocols explained in Materials and Methods. The virulence genes studied were *exoS* (A), *lasA* (B), *pcrV* (C), *plcB* (D), *pilM* (E), and *pscH* (F). The results of the virulence genes are always shown compared to the expression of strain PAO1 (set at 1). Values are means plus SD from independent experiments performed as described in Materials and Methods. The values that are significantly different (*P* < 0.05 by Student’s *t* test) are indicated by a bar and asterisk.

To confirm and expand these results, we next analyzed the expression of six key selected virulence genes (*lasA*, *plcB*, *pilM*, *pcrV*, *pscH*, and *exoS* or *exoU*) through reverse transcription-PCR (RT-PCR) in wild-type strains PAO1 (Fig. 7) and PA14 (see Fig. S5 in the supplemental material) and their respective mutant derivatives. As can be observed, downregulation of all these genes was confirmed for the PAO1 *ampD* triple mutant (Fig. 7) and also evidenced for the PA14 *ampD* triple mutant using in this case *exoU* instead of *exoS* (Fig. S5). Moreover, as mentioned above, inactivation of *ampC* and *trans*-complementation with a wild-type *ampD* gene restored wild-type expression. Likewise, overexpression of *ampC* caused by *ampD dacB* inactivation did not modify virulence gene expression, while the *ampD ampDh3* double amidase mutant had only a modest effect on expression of some of the virulence genes (Fig. 7; Fig. S5).

### Dissection of the underlying basis for the pathway-dependent impact of *ampC* derepression on fitness and virulence.

**FIG 8:**
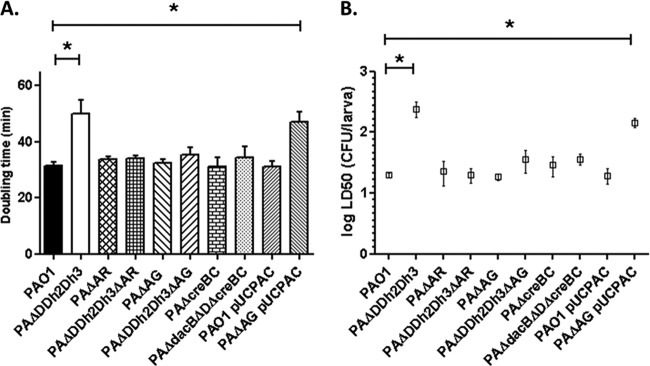
(A) Duplication times of *P*. *aeruginosa* strains. Duplication times (in minutes) of exponentially growing cells of strain PAO1 and strains derived from strain PAO1 in LB broth are shown. Values are means ± SD from three independent experiments performed for each strain. The values that are significantly different (*P* < 0.0001 by Student’s *t* test) from the value for the wild-type strain are indicated by a bar and asterisk. (B) *Galleria mellonella* killing assays with PAO1-derived strains. *G. mellonella* larvae were infected as described in Materials and Methods. LD_50_s were determined as previously described (53). To analyze the differences between the obtained LD_50_s, Student’s *t* test was performed with the values of the three independently obtained LD_50_s. The values that are significantly different (*P* < 0.005 by Student’s *t* test) from the value for the wild-type strain are indicated by a bar and asterisk.

Results obtained so far clearly indicated that very high-level *ampC* expression (>1,000-fold compared to the wild-type value) has a major impact on fitness and virulence, but only if occurring in an amidase-deficient background. Since amidase activity is required for peptidoglycan turnover, we hypothesized that the observed effect could be caused by overexpressing *ampC* in the absence of cell wall recycling. To test this hypothesis, we first analyzed the individual effect of blocking of peptidoglycan recycling by using an *ampG* mutant of strain PAO1 and the individual effect of *ampC* overexpression by introducing into PAO1 a plasmid-encoded *ampC* gene (pUCPAC), providing expression levels similar to that of the *ampD* triple mutant (approximately 1,500-fold increased expression [Table 1]). Indeed, as can be observed in Fig. 8, neither inactivation of *ampG* nor overexpression of a plasmid-mediated *ampC* significantly modified fitness (doubling time) or virulence (*G. mellonella* lethality) of strain PAO1. However, when both features were gathered together in the PAO1 *ampG* mutant expressing the plasmid-mediated *ampC* gene, a major effect on fitness and virulence was evidenced (Fig. 8), proving the established hypothesis.

Moreover, other potential hypotheses that could have eventually explained the different behavior of the *ampD* triple mutant compared to other mutants showing similar levels of AmpC expression (such as the *ampD dacB* double mutant) were ruled out. These hypotheses included a potential differential effect on *ampR* expression, which was ruled out since the transcription of this gene was not modified in any of the mutants (see Fig. S6 in the supplemental material). Besides, as expected, knocking down *ampG* or *ampR* in the triple amidase mutant abolished *ampC* overexpression and consequently restored wild-type fitness and virulence phenotype (Fig. 8). Finally, we also ruled out the possibility that the preservation of fitness and virulence of the *dacB* mutants was driven by the previously documented activation of the *creBC* system (28), since knocking down this two-component regulatory system did not produce a significant effect on fitness and virulence (Fig. 8).

## DISCUSSION

In this work, we show that the lack of the three *P. aeruginosa* N-acetyl-anhydromuramyl-l-alanine amidases causes a dramatic effect in fitness, severely compromising growth rates and bacterial motility (twitching, swimming, and swarming). Moreover, cytotoxicity, but not invasiveness, is specifically impaired in the triple amidase mutant, likely due to the repression of key virulence factors such as the protease LasA, phospholipase C, or TTSS components. Moreover, the inactivation of the three amidases in both PAO1 and PA14 strains significantly increased LD_50_s in the *G. mellonella* model. It is noteworthy that the higher pathogenicity of strain PA14 compared to the pathogenicity of strain PAO1 (30) was fully abolished upon disruption of *N*-acetyl-anhydromuramyl-l-alanine amidase activity; in other words, while LD_50_s were much lower for strain PA14 than for strain PAO1, the LD_50_s of the corresponding amidase triple mutants were much higher but nearly identical in both mutants. This is likely explained by the fact that the higher virulence of PA14 mainly relies on its potent cytotoxicity (related to ExoU production and an elevated TTSS activity [30, 31]), and cytotoxicity is specifically impaired in the triple amidase mutant. In other words, although the reduced growth rates may largely explain the reduced pathogenicity, the specific impairment of cytotoxicity is also very relevant for the virulence-attenuated phenotype, especially in the case of PA14.

Likely, the most simple explanation for the observed GMC phenotype would be that the very high level of *ampC* expression determines a major energetic burden compromising cell physiology and, consequently, fitness and virulence. Indeed, this hypothesis would be supported by the fact that knocking out *ampC* in the *ampD* triple mutant fully restored the wild-type phenotype. However, the level of *ampC* overexpression by itself does not explain the GMC phenotype, since similar levels are also produced through alternative pathways, such as the double inactivation of *ampD* and *dacB* genes, which does not affect fitness or virulence. Thus, so far we could conclude that the very high level of *ampC* overexpression was required but not sufficient for the observed GMC phenotype. Alternatively to the hypothesis of energetic burden of *ampC* expression, the activity of AmpC itself could have an effect on cell physiology. Indeed, AmpC and other β-lactamases may modify the structure of the peptidoglycan, due to residual dd-peptidase activity reminiscent of their PBP ascendance (32, 33). In any case, activity of AmpC by itself would still be necessary but not sufficient to produce the GMC phenotype, since as discussed above, alternative paths leading to similar levels of *ampC* overexpression do not show altered fitness or virulence. Thus, our data suggest that *ampC* overexpression and the peptidoglycan alterations produced by the specific mutations should act in concert to confer the observed GMC phenotype.

We have previously shown that *ampC* overexpression driven by both *ampD* and *dacB* inactivation requires a functional AmpG and an activated AmpR (22, 34). Thus, although there is evidence supporting the hypothesis that AmpG or AmpR may play a role in virulence (4, 27, 35), they do not affect differentially both *ampC* overexpression pathways. Moreover, transcription of *ampR* was not affected in any of the mutants.

AmpD, AmpDh2, and AmpDh3 are the three *N*-acetyl-anhydromuramyl-l-alanine amidases of *P. aeruginosa*. AmpD is the cytoplasmic enzyme processing soluble 1,6-anhydromuropeptides for the recycling of peptidoglycan catabolites and avoiding *ampC* induction (36). AmpDh2 and AmpDh3 are periplasmic enzymes mainly acting directly on the insoluble polymeric sacculus of the peptidoglycan for cell wall remodeling (36, 37). However, they can also process soluble 1,6-anhydromuropeptides but with a lower efficiency than that for AmpD (36–38). Thus, candidates for triggers of the fitness and virulence impairment in the amidase triple mutant would be the accumulation of soluble 1,6-anhydromuropeptides and/or the alteration of cell wall remodeling itself. Indeed, cell wall remodeling has been shown to be relevant for the assembly of flagella and for type III and type VI secretion systems (39–42). Moreover, although our results related to membrane physiology (susceptibility to serum, neutrophil killing, and electron microscopy) failed to reflect any differences between the triple AmpD mutant and wild-type, the possibility of AmpDh2 and/or AmpDh3 deletion affecting to a certain degree the assembly and anchoring of bacterial surface structures cannot be completely ruled out and should be explored further, given the existing evidences in this context (43–45).

As mentioned above, a similar (around 1,000-fold) *ampC* expression level in the *dacB ampD* double mutant does not produce a significant fitness or virulence effect. Accumulation of soluble 1,6-anhydromuropeptides should however also be high and similar to the amidase triple mutant, since cytosolic AmpD is, as commented above, the main factor. Moreover, the absence of *dacB*-encoded PBP4 should also further increase muropentapeptide levels (25). Still, an obvious qualitative difference between the *ampD* triple mutant and the other mutants is the lack of *N*-acetyl-anhydromuramyl-l-alanine amidase activity in the former, leading to a complete blocking of peptidoglycan recycling. Indeed, our experiments using an *ampG* mutant of strain PAO1 and a plasmid-mediated *ampC* demonstrated that fitness-virulence impairment is produced by the blocking of peptidoglycan recycling and simultaneous *ampC* overexpression.

Finally, we also ruled out the alternative hypothesis that the preservation of fitness and virulence of the *dacB* mutants was driven by the previously documented activation of the *creBC* system (shown to play a major role in fitness and response to β-lactam challenge [22, 26]), since knocking out this two-component regulatory system did not produce a significant effect on fitness and virulence.

Regardless of the underlying mechanisms, our results are helpful to understand the natural occurrence of AmpC-hyperproducing mutants among clinical strains, since while *ampD dacB* double mutants have been well documented (22, 34), *ampD* triple mutants have not been reported so far. Another practical lesson from this work is that blocking peptidoglycan recycling (e.g., using AmpG inhibitors) might not only be a useful strategy for combating inducible (AmpR-dependent) β-lactam resistance (46, 47) but also AmpR-independent plasmid-mediated AmpCs, frequently seen in members of the family *Enterobacteriaceae* (48).

In summary, altogether, our results determine a major step forward for understanding bacterial β-lactam resistance responses and should be helpful for guiding the development of future strategies for combating *P. aeruginosa* infections.

## MATERIALS AND METHODS

### Bacterial strains and plasmids.

A list and description of the bacterial strains and plasmids used in this work are shown in Table S2 in the supplemental material. *P. aeruginosa* single or combined knockout *ampD*, *ampDh2*, *ampDh3*, *ampC*, or *dacB* mutants were constructed according to previously described procedures (20, 22) based on the Cre-lox system for gene deletion in *P. aeruginosa* (49) and confirmed by DNA sequencing. Since *ampR* is contiguous to *ampC*, the absence of altered *ampR* sequence and expression in the *ampC* mutants was confirmed through DNA sequencing and transcriptional analysis through real-time reverse transcription-PCR (RT-PCR), respectively. The previously constructed plasmid pUCPAD was used for the complementation of selected knockout mutants through electroporation followed by selection in Müller-Hinton agar plates containing 50 mg/liter gentamicin (9).

### Global transcriptome analysis.

Three independent replicates of *P*. *aeruginosa* strains PAO1 and PAΔDDh2Dh3 (PAO1 with *ampD ampDh2 ampDh3* knocked out) were grown at 37°C to an optical density at 600 nm (OD_600_) of 1 in vigorously shaken 50-ml flasks containing 10 ml of LB broth. The total RNA was isolated using the RNeasy minikit (Qiagen), according to the manufacturer’s instructions. RNA was treated with 2 U of Turbo DNase (Ambion) to remove DNA. Ten micrograms of total RNA was used for cDNA synthesis, fragmentation, labeling, and hybridization according to the Affymetrix GeneChip *P. aeruginosa* genome array expression analysis protocol. Briefly, random hexamers (Invitrogen) were added to the 10 µg of total RNA, and *in vitro*-synthesized, polyadenylated transcripts for *Bacillus subtilis* genes were used as a control as recommended. cDNA was synthesized using Superscript II (Invitrogen) according to the manufacturer’s instructions. The reaction was inactivated at 70°C for 10 min, and after hydrolysis with NaOH and neutralization with HCl, cDNA was purified by using a High Pure PCR product purification kit (Roche). The cDNA was fragmented by DNase I (Thermo Fisher Scientific), and the final 3′ end labeling of the fragmentation products was performed with the GeneChip DNA labeling reagent (Affymetrix) following the recommended Affymetrix protocol.

Arrays were hybridized following the manufacturer’s (Affymetrix) instructions. Signal values were generated by the Affymetrix Expression Console 1.4.1 software program, and normalization of the signal values was performed using the Robust Multichip Analysis (RMA) algorithm (50). Statistical analysis was executed by the Transcriptome Analysis Console 3.0 software (Affymetrix) as described previously (22). Only transcripts showing ≥2-fold increases or decreases and one-way analysis of variance (ANOVA) *P* values less than 0.05 were considered to be differentially expressed. In all cases, the posterior probability for differential expression (PPDE) was between 0.999 and 1. The expression of selected representative genes was further analyzed using real-time RT-PCR.

### Analysis of gene expression.

The relative mRNA levels of selected genes (*ampC*, *exoU*, *exoS*, *lasA*, *pcrV*, *plcB*, *pilM*, and *pscH*) were determined by real-time RT-PCR, according to previously described protocols (10, 20). Total RNA was obtained with the RNeasy minikit (Qiagen). Fifty nanograms of purified RNA was used for one-step reverse transcription and real-time PCR using an Illumina Eco real-time PCR system as previously described (20). The *rpsL* housekeeping gene was used to normalize the expression levels, and the results were always referred to PAO1 or PA14. All RT-PCRs were performed in duplicate, and the mean values of expression from three independent experiments were considered. The primers used are listed in Table S3 in the supplemental material.

### Motility assays.

The swimming, swarming, and twitching motilities were determined in the selected strains as described previously (51) in plates containing different media. (i) To determine swimming motility, 10 g/liter tryptone, 5 g/liter NaCl, and 0.3% (wt/vol) mid-resolution agarose was used. The plates were inoculated with an isolated colony from an overnight culture in LB agar at 37°C, using a sterile toothpick. (ii) To determine swarming motility, 1× M8 minimal medium supplemented with 1 mM MgSO_4_, 0.2% glucose, 0.5% Bacto Casamino Acids, and 0.5% agar. Aliquots (2.5 µl) were taken from overnight cultures to inoculate the surface of the plate. (iii) To determine twitching motility, isolated colonies were inoculated with a sterile toothpick inserted in the bottom of LB agar plates. In all cases, the plates were wrapped with film to prevent dehydration and incubated at 37°C for 16 h. After incubation, the diameter of the motility zone was measured. In the plates used to determine twitching motility, the medium was taken off the plate, and the print over the dish bottom was measured. If the area was irregular, two perpendicular diameters were measured, and the result was expressed as the mean. Ten determinations for each strain and motility type were recorded.

### Invertebrate infection model.

The wax moth *Galleria mellonella* was used as infection model following previously described protocols (29, 30, 52). Exponentially growing cultures were pelleted, washed, and resuspended in phosphate-buffered saline (PBS). Different serial dilutions (depending on the strain) were made in PBS. A Hamilton syringe was used to inject 10-µl aliquots into individual fifth-instar *G. mellonella* larvae via the hindmost left proleg. Ten larvae were injected for each dilution and strain, and larvae were scored as live or dead after 24 h at 37°C. An approximate 50% lethal dose (LD_50_) was initially determined in a pilot screening of wide bacterial load intervals (logarithmic scale). When an approximate LD_50_ was obtained, three final experiments with already adjusted bacterial loads were performed. In all cases, 10 larvae were inoculated with 10 µl of PBS as controls. The percentage of larvae that had died at each bacterial dose was then analyzed by Probit analysis (53), and the LD_50_ ± standard deviation was finally determined using R software, version 3.2.2.

### Data analysis.

With the exception of LD_50_s (see above), GraphPad Prism 5 software was used for graphical representation and statistical analysis. Quantitative variables were compared using Student’s *t* test or Mann-Whitney U test as appropriate. A *P* value of <0.05 was considered statistically significant.

An extended Materials and Methods section is available in the supplemental material (Text S1 in the supplemental material).

## SUPPLEMENTAL MATERIAL

Text S1  Supporting Materials and Methods. Download Text S1, DOCX file, 0.02 MB

Figure S1  Growth curves of representative strains derived from strain PAO1. The growth curves were determined as described in Materials and Methods. Experiments were performed on at least three independent occasions; the symbols represent the means of the three replicate values, and the standard deviations (SD) are represented by the error bars. Download Figure S1, TIF file, 0.2 MB

Figure S2  Electron microscopy pictures of PAO1 and PAΔDDh2Dh3 strains. Negative staining (A and B), SEM (C and D), and TEM (E and F) images showed no differences between strains PAO1 (A, C, and E) and PAΔDDh2Dh3 (B, D, and F) regarding the presence and width of flagella, general appearance of the cell surface, or the width and structure of the bacterial envelopes. Download Figure S2, TIF file, 1.5 MB

Figure S3  Complement-mediated and neutrophil-mediated killing of PAO1 and PA14 strains and mutants derived from these two strains. (A) Percentage of bacterial survival after incubation with NHS with regard to samples incubated with HI-NHS. The displayed results represent the means ± SDs from at least three independent experiments. (B and C) Percentage of bacterial survival after incubation of wild-type strains PAO1 (B) and PA14 (C) and mutants derived from these two strains with neutrophils (MOI of 5:1) for 1 h, with 10% NHS or HI-NHS. The bars represent the mean (plus SD) percentage of surviving bacteria with respect to the initial inoculum. Download Figure S3, TIF file, 0.3 MB

Figure S4  IL-8 response of A549 cells after infection with *P. aeruginosa* strains or stimulation with cell-free supernatants or purified peptidoglycans (PGNs). IL-8 secretion in RPMI 1640 medium during stimulation with intact *P. aeruginosa* strains (MOI of 100; 3 h) (A), RPMI 1640 medium containing 10% cell-free culture (20 h) (B), or 2.5 µg/ml of purified PGNs (20 h) (C). The control values correspond to the basal level of IL-8 release without any stimuli (3 h or 20 h). The results represent the mean ± SD from at least three wells of A549 cells from three independent plates. Download Figure S4, TIF file, 0.4 MB

Figure S5  Expression of selected virulence genes in PA14-derived strains, determined through RT-PCR, following the protocols described in Materials and Methods. The virulence genes were *exoU* (A), *lasA* (B), *pcrV* (C), *plcB*(D), *pilM* (E), and *pscH* (F). The results are always relative to the expression of wild-type PA14 (set at 1). The results represent the means ± SD from the independent experiments conducted as described in Materials and Methods. *, *P* < 0.05 by Student’s *t* test. Download Figure S5, TIF file, 0.5 MB

Figure S6  Expression of transcriptional regulator AmpR in selected strains determined through RT-PCR, following the protocols described in Materials and Methods. The results are always relative to the expression of wild-type PAO1 (set at 1). The results represent the means ± SD from the independent experiments conducted as described in Materials and Methods. *, *P* < 0.05 by Student’s *t* test. Download Figure S6, TIF file, 0.2 MB

Table S1  Genes up- or downregulated in the triple *ampD* mutant compared to wild-type PAO1 strain. Table S1, DOCX file, 0.02 MB

Table S2  Strains and plasmids used in this work. Table S2, DOCX file, 0.02 MB

Table S3  Primers used in this study. Table S3, DOCX file, 0.02 MB
