# Supplementary material for: Impact of AmpC Derepression on Fitness and Virulence: the Mechanism or the Pathway?
Source: mBio. 2016 Oct 25;7(5):e01783-16. doi: 10.1128/mBio.01783-16 (PMC5080387; doi:10.1128/mBio.01783-16)
Supplement: Text S1 — Supporting Materials and Methods. Download [file mbo005163047s1.docx]

**SUPPLEMENTAL MATERIAL**

**Text S1: SUPPORTING MATERIALS AND METHODS**

**Antibiotic susceptibility testing.** In wild-type PAO1 and PA14 strains and their respective mutant derivatives , the minimal inhibitory concentrations (MICs) of Imipenem, Meropenem, Piperacillin-Tazobactam, Ceftazidime, Cefepime, Aztreonam and Colistin were determined by E-test (BioMeriéux), whereas MICs of vancomycin were determined by microdilution on Müller-Hinton broth.

**In vitro growth rates**. For growth rate experiments, 1 ml samples of overnight cultures from the studied strains were diluted in 50 ml of fresh LB broth in 250 mL flasks, and incubated at 37°C and 180 rpm. The growth curves were obtained measuring optical density at 600 nm each 30 minutes, until the cultures reached the stationary phase. In addition, the doubling times of exponentially growing cells were determined by plating serial dilutions on LB agar plates at 1h intervals. At least three independent experiments were performed for each of the selected strains.

**Electron microscopy.** i) Negative staining: *P. aeruginosa* strains were grown on LB agar plates for 16 h and resuspended in 0.1M phosphate buffer. An aliquot was added to a grid coated with a Formvar supporting film coated with carbon for 5 min. The grid was stained with 2% uranyl acetate in water for 5 min, rinsed in distilled water and air-dried. Samples were visualized in a Tecnai FEI 10 electron microscope at 80 kV. Digital images were taken by using a Veleta camera (Olympus Soft Imaging Solutions). ii) Transmission electron microscopy (TEM): *P. aeruginosa* cells, prepared as described above were fixed in a 2% glutaraldehyde in 0.1 M sodium cacodylate buffer and 0.1 M sucrose + 3 mM CaCl_2_, pH 7.4. Then were rinsed in 0.1 M phosphate buffer, followed by post fixation in 2% osmium tetroxide in 0.1 M PB at +4°C for 2 h. Then, samples were dehydrated by ethanol acetone treatment and embedded in LX-112 (Ladd). Ultrathin sections (50–60 nm) were cut by a Leica EM UC 6 (Leica)and contrasted with uranyl acetate followed by lead citrate and examined in a Tecnai 12 Spirit Bio TWIN TEM (FEI Company) at 100 kV. Digital images were taken by using a Veleta camera (Olympus Soft Imaging Solutions). iii) Scanning electron microscopy (SEM): specimens were fixed as described above. The specimens were transferred to a NL 16 polyamide filter (Whatman) and briefly rinsed in distilled water and dehydrated by ethanol-acetone treatment. They were then dried in a critical point dryer (Bal-Tec) using carbon dioxide. Specimens were mounted on an Aluminum stub and coated with Carbon (Bal-Tec). The specimens were analyzed in an Ultra 55 field emission scanning electron microscope (Zeiss) at 3 kV.

**Serum bacterial killing.** Serum bactericidal assays were performed through previously described protocols (1) with slight modifications. Bacteria from overnight LB agar plates were resuspended in PBS with Ca and Mg (Sigma) to a final concentration of approximately 2.5x10^7^ CFU/mL; 80 µl of this suspension were mixed with 40 µl of PBS with Ca and Mg and 40 µl of nonimmune human serum (NHS) or heat-inactivated (HI) NHS. The samples were incubated at 37°C for 30 min and then serially diluted and plated to determine the bacterial killing with regards to the number of bacteria in tubes with HI-NHS. Experiments were always performed at least in triplicate.

**Neutrophil bacterial killing**. Fresh neutrophils from healthy donors not immunized against *P. aeruginosa*, were obtained as previously described (2). After elimination of serum, lymphocytes and monocytes through Ficoll centrifugation, the red blood cells were lysed. The remaining white cells pellet obtained after centrifugation (overwhelmingly corresponding to neutrophils) was then washed with PBS and resuspended with RPMI to a final concentration of approximately 1E^7^neutrophils /mL. To determine the neutrophil-mediated killing of *P. aeruginosa*, the method described by Dacheux et al was performed with slight modifications (3). Briefly, approx. 2x10^6^ neutrophils were co-incubated with 1x10^7^ CFU of each bacterial strain (Multiplicity Of Infection, MOI, of 5) in a final volume of 500 µl of RPMI with 10% of NHS or HI-NHS, during 1 hour at 37ºC in a rotation incubator. Neutrophils were lysed by the addition of 1 µl of Triton X-100, and samples were serially diluted and plated to determine the number of viable bacteria. Platings in the beginning of incubation were also done to quantify the initial number of bacteria. Experiments were always performed at least in triplicate.

**Peptidoglycan purification.** The PGN from selected mutants were extracted following previously described protocols with slight modifications (4, 5). PAO1, PA14 and derived mutants were grown overnight in 1L of LB broth at 37°C and 180 rpm. The cells were resuspended in double-distilled water. An equal volume of boiling 20% SDS solution was slowly added, and the final suspension was kept boiling for 12 h with stirring. The suspensions were centrifuged at 18000 g for 45 min to collect the sacculi fraction, which was then washed with warm sterile double-distilled water at least three times. PGNs were suspended in 10 mM Tris-HCl (pH 7.6) supplemented with 0.5 mM CaCl_2_ and 2.5 mM MgCl_2_, and treated with α-amylase (Sigma-Aldrich), Turbo DNAse (Ambion), RNAse (Sigma-Aldrich) for 2 h at 37°C, and finally with pronase E (Merck) at 60°C for 90 min. The enzymes were inactivated and next, PGNs were collected and washed as described above. After that, PGNs were lyophilized for weighing and quantification. Samples were treated with 8 M LiCl for 1 h at room temperature. The PGNs were centrifuged and washed three times, and treated with 100 mM EDTA for 1 h at room temperature. Samples were centrifuged and washed as above, and treated with acetone for 1 h at room temperature. After at least three washes, the pellets were resuspended in 50 mM NaH_2_PO_4_ (pH 4.5) and digested with mutanolysin (Sigma-Aldrich) overnight. Next, the enzyme was inactivated and the samples were centrifuged for 5 min to remove insoluble debris. Finally, the supernatants were 0.22 µm-filtered. The E-toxate reagent (Sigma-Aldrich) was used to check the absence of endotoxin contamination.

**Cell-free bacterial supernatants preparation and inactivation of bacteria.** Supernatants proceeding from overnight LB cultures were adjusted to an OD_600_=2 with fresh LB, centrifuged and 0.22 µm-filtered. For bacteria inactivation, aliquots of overnight LB cultures were taken to have an approximate number of 5x10^6^ CFUs. The samples were then centrifuged and resuspended with 20 µL of PBS, and incubated for 10 min at 96ºC.

**Cell culture, invasion and cytotoxicity assays.** The A549 human type II alveolar epithelial cell line was purchased from Cell Line Service (Germany) and used between the passages 3 and 30. The cells were maintained in Dulbecco’s modified Eagle’s medium (DMEM) (Sigma-Aldrich) supplemented with 10% of heat-inactivated fetal bovine serum, 10mM HEPES, 2 mM L-glutamine and 1X antibiotic-antimycotic solution (Biowest). Cells were seeded at approx.1x10^5^ cells per well in 24 well plates the day before experiments. The day after, the cells were at approx. 80% of confluence, and were infected at a MOI of 100, following previously described protocols (6): bacteria were grown to log phase , and bacteria were diluted in RPMI 1640 medium (Biowest). When indicated, the plates were centrifuged (1000 g for 5 min) to synchronize the arrival of bacteria to the cells. After incubation of 3 hours, the medium was collected and stored at -80ºC for additional analysis. When the strains were derivatives of PA14 (cytotoxic strain), the cells were discarded. On the other hand, when PAO1 (invasive strain) derivatives were used, the protocol was completed to determine the invasion: the medium containing the bacteria was stored and replaced with fresh RPMI 1640 with 0.4 mg/ml of amikacin. The plates were incubated for 1 hour to kill extracellular bacteria. The media was removed and cells washed with PBS. 0.5 mL of PBS containing 0,1% of Triton X-100 were added to each well, and incubated for 10 min to release the bacteria. Serial dilutions were plated in duplicate to determine the number of CFUs . The supernatants of the cited infection assays were used for IL-8 release (next paragraph) and cytotoxicity determinations. For this last purpose, the Cytotoxicity Detection Kit PLUS (Roche) was used, following manufacturer’s instructions.

Additionally, the purified PGNs and cell-free bacterial supernatants were used as stimuli over the A549 cultures, to assess the release of IL-8 / LDH. The purified PGNs were added at 2.5 µg/mL in RPMI medium, and incubated for 20 hours. The cell-free bacterial growth supernatants were diluted in RPMI medium to a final concentration of 10%, and incubated for 20 h. In these stimulations, the A549 cells were used at 50% of confluency, and the mentioned determinations were performed with samples proceeding from at least 9 wells (three wells from each of 3 independent plates) per strain and condition.

**Inflammatory response.** To assess the inflammatory response elicited by the mentioned stimuli over the A549 cell cultures, the secretion of IL-8 interleukin was used as indicator (6). The supernatants of cells after stimulation were used as samples and samples of cells without any stimuli were used as basal controls. The Human IL-8/NAP-1 Instant ELISA kit (eBioscience-Affymetrix) was used following the manufacturer’s instructions.

**Text S1 References**

1. Martínez-Ramos I, Mulet X, Moyá B, Barbier M, Oliver A, Albertí S. 2014. Overexpression of MexCD-OprJ reduces *Pseudomonas aeruginosa* virulence by increasing its susceptibility to complement-mediated killing. Antimicrob Agents Chemother 58:2426-2429.PMID: 24419345.
2. Martinez-Serra J, Robles J, Nicolàs A, Gutierrez A, Ros T, Amat JC, Alemany R, Vögler O, Abelló A, Noguera A, Besalduch J. 2014. Fluorescence resonance energy transfer-based real-time polymerase chain reaction method without DNA extraction for the genotyping of F5, F2, F12, MTHFR, and HFE. J Blood Med 5:99-106. PMID: 25028568.
3. Dacheux D, Attree I, Schneider C, Toussaint B. 1999. Cell death of human polymorphonuclear neutrophils induced by a *Pseudomonas aeruginosa* cystic fibrosis isolate requires a functional type III secretion system. Infect Immun 67:6164-7.
4. Ropy A, Cabot G, Sánchez-Diener I, Aguilera C, Moya B, Ayala JA, Oliver A . 2015. Role of *Pseudomonas aeruginosa* low-molecular-mass penicillin-binding proteins in AmpC expression, β-lactam resistance, and peptidoglycan structure. Antimicrob Agents Chemother 59: 3925-3934. PMID: 25896695.
5. Zhang W, Lee M, Hesek D, Lastochkin E, Boggess B, Mobashery S. 2013. Reactions of the three AmpD enzymes of *Pseudomonas aeruginosa*. J Am Chem Soc 135: 4950-4953. PMID: 23510438.
6. Hawdon NA, Aval PS, Barnes RJ, Gravelle SK, Rosengren J, Khan S, Ciofu O, Johansen HK, Høiby N, Ulanova M. 2010. Cellular responses of A549 alveolar epithelial cells to serially collected *Pseudomonas aeruginosa* from cystic fibrosis patients at different stages of pulmonary infection. FEMS Immunol Med Microbiol 59:207-220. PMID: 20528926.
