# Supplementary figures and images for: Impact of AmpC Derepression on Fitness and Virulence: the Mechanism or the Pathway?
Source: mBio. 2016 Oct 25;7(5):e01783-16. doi: 10.1128/mBio.01783-16 (PMC5080387; doi:10.1128/mBio.01783-16)

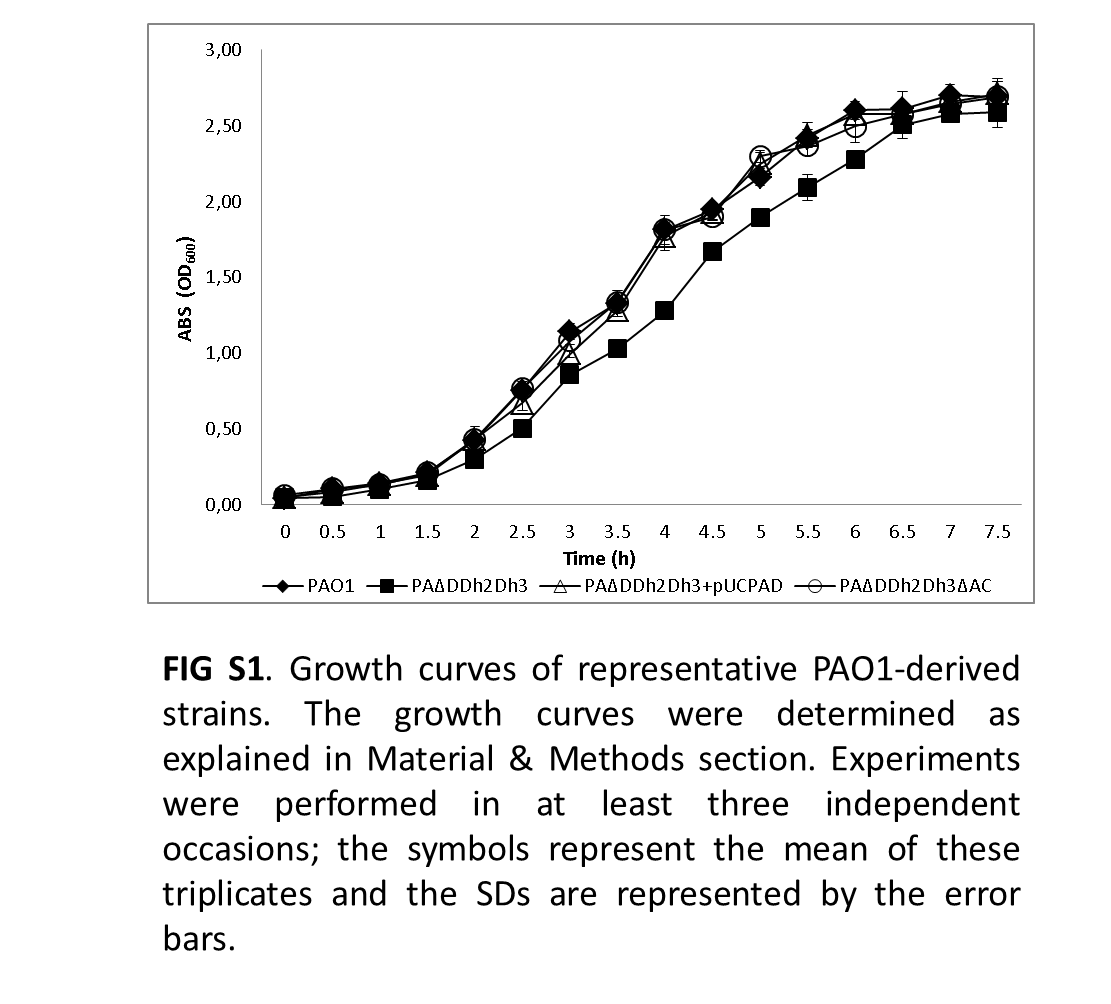

Supplement: Figure S1 — Growth curves of representative strains derived from strain PAO1. The growth curves were determined as described in Materials and Methods. Experiments were performed on at least three independent occasions; the symbols represent the means of the three replicate values, and the standard deviations (SD) are represented by the error bars. Download [file mbo005163047sf1.tif]

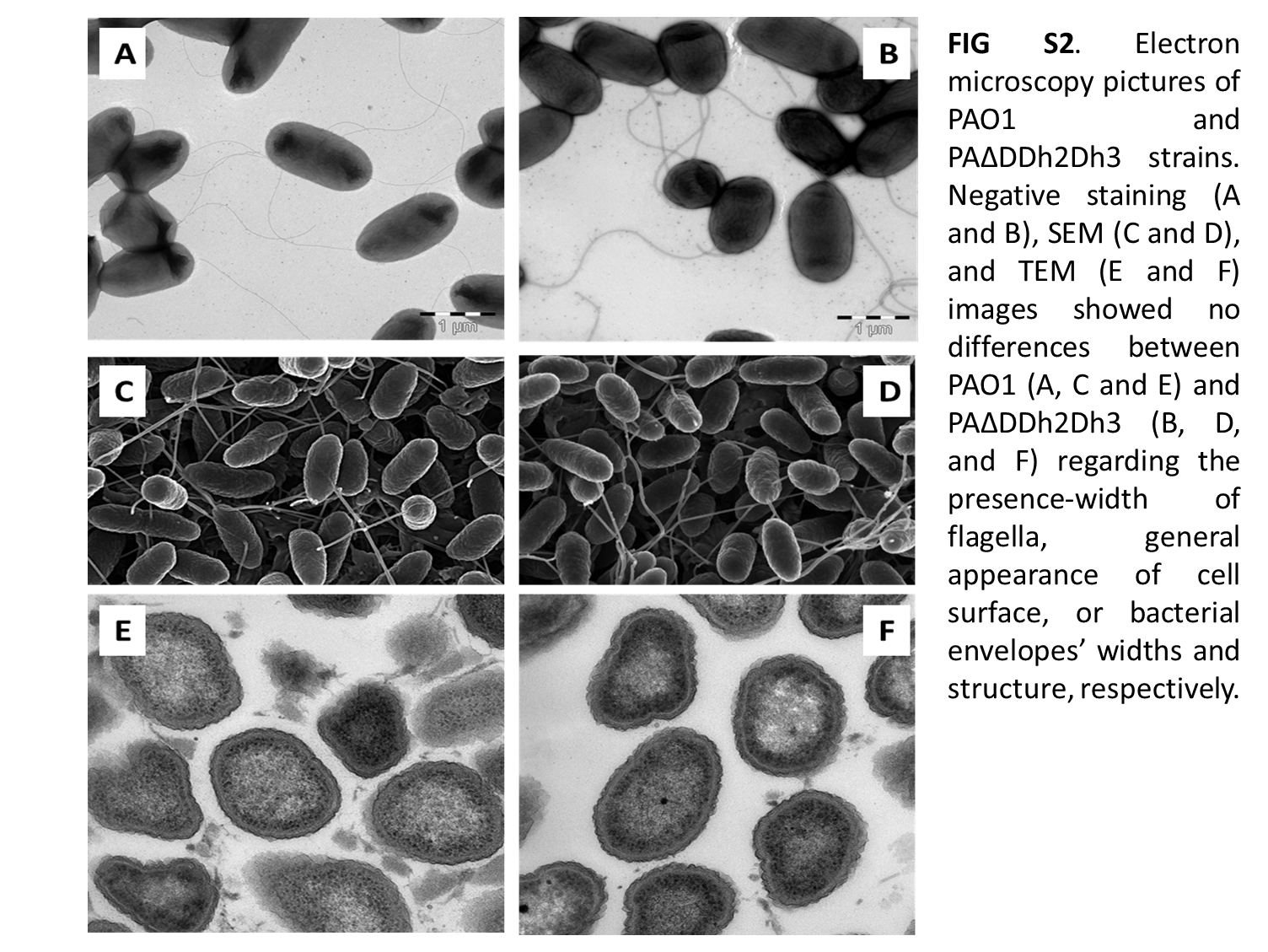

Supplement: Figure S2 — Electron microscopy pictures of PAO1 and PAΔDDh2Dh3 strains. Negative staining (A and B), SEM (C and D), and TEM (E and F) images showed no differences between strains PAO1 (A, C, and E) and PAΔDDh2Dh3 (B, D, and F) regarding the presence and width of flagella, general appearance of the cell surface, or the width and structure of the bacterial envelopes. Download [file mbo005163047sf2.tif]

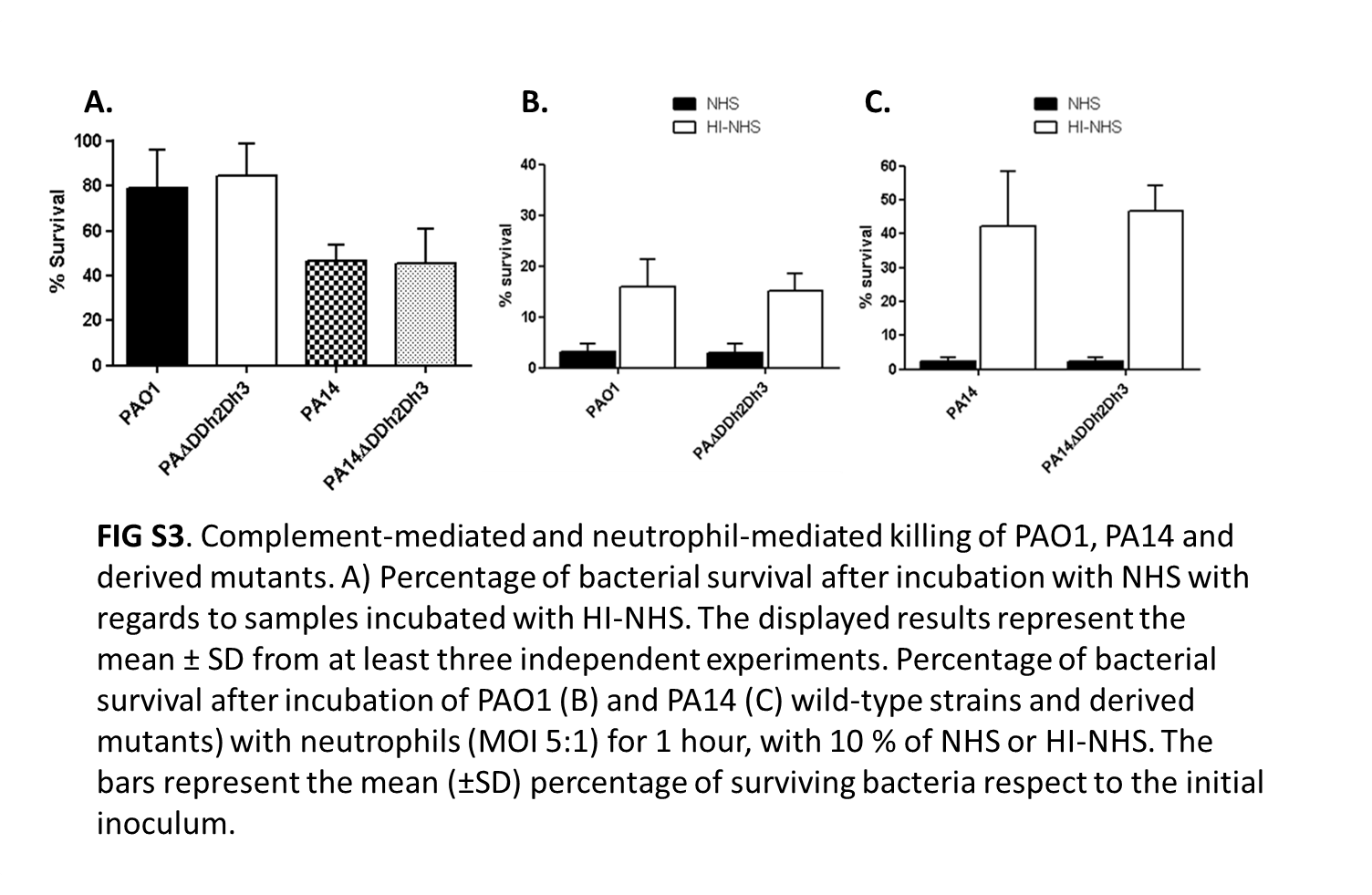

Supplement: Figure S3 — Complement-mediated and neutrophil-mediated killing of PAO1 and PA14 strains and mutants derived from these two strains. (A) Percentage of bacterial survival after incubation with NHS with regard to samples incubated with HI-NHS. The displayed results represent the means ± SDs from at least three independent experiments. (B and C) Percentage of bacterial survival after incubation of wild-type strains PAO1 (B) and PA14 (C) and mutants derived from these two strains with neutrophils (MOI of 5:1) for 1 h, with 10% NHS or HI-NHS. The bars represent the mean (plus SD) percentage of surviving bacteria with respect to the initial inoculum. Download [file mbo005163047sf3.tif]

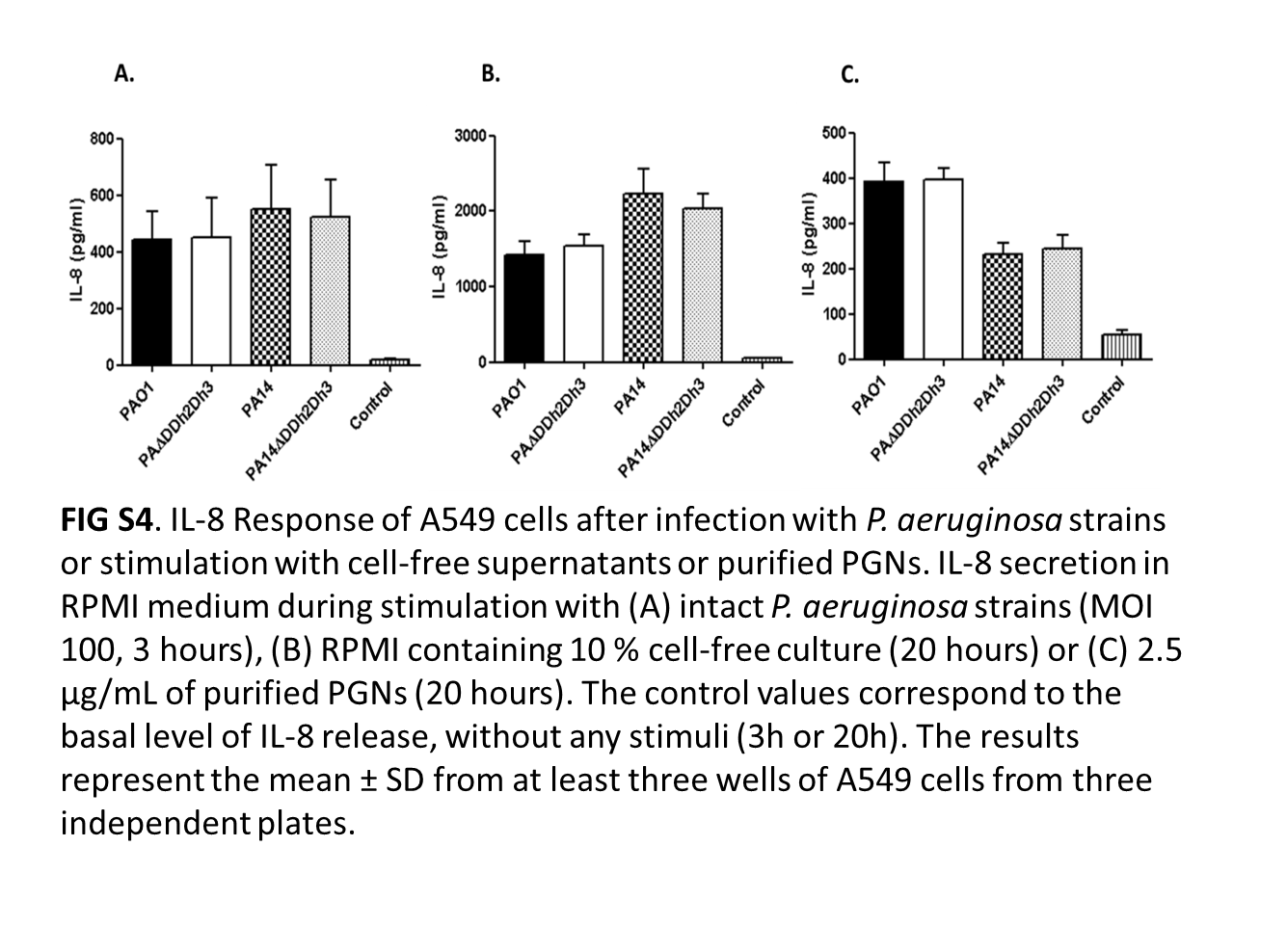

Supplement: Figure S4 — IL-8 response of A549 cells after infection with P. aeruginosa strains or stimulation with cell-free supernatants or purified peptidoglycans (PGNs). IL-8 secretion in RPMI 1640 medium during stimulation with intact P. aeruginosa strains (MOI of 100; 3 h) (A), RPMI 1640 medium containing 10% cell-free culture (20 h) (B), or 2.5 µg/ml of purified PGNs (20 h) (C). The control values correspond to the basal level of IL-8 release without any stimuli (3 h or 20 h). The results represent the mean ± SD from at least three wells of A549 cells from three independent plates. Download [file mbo005163047sf4.tif]

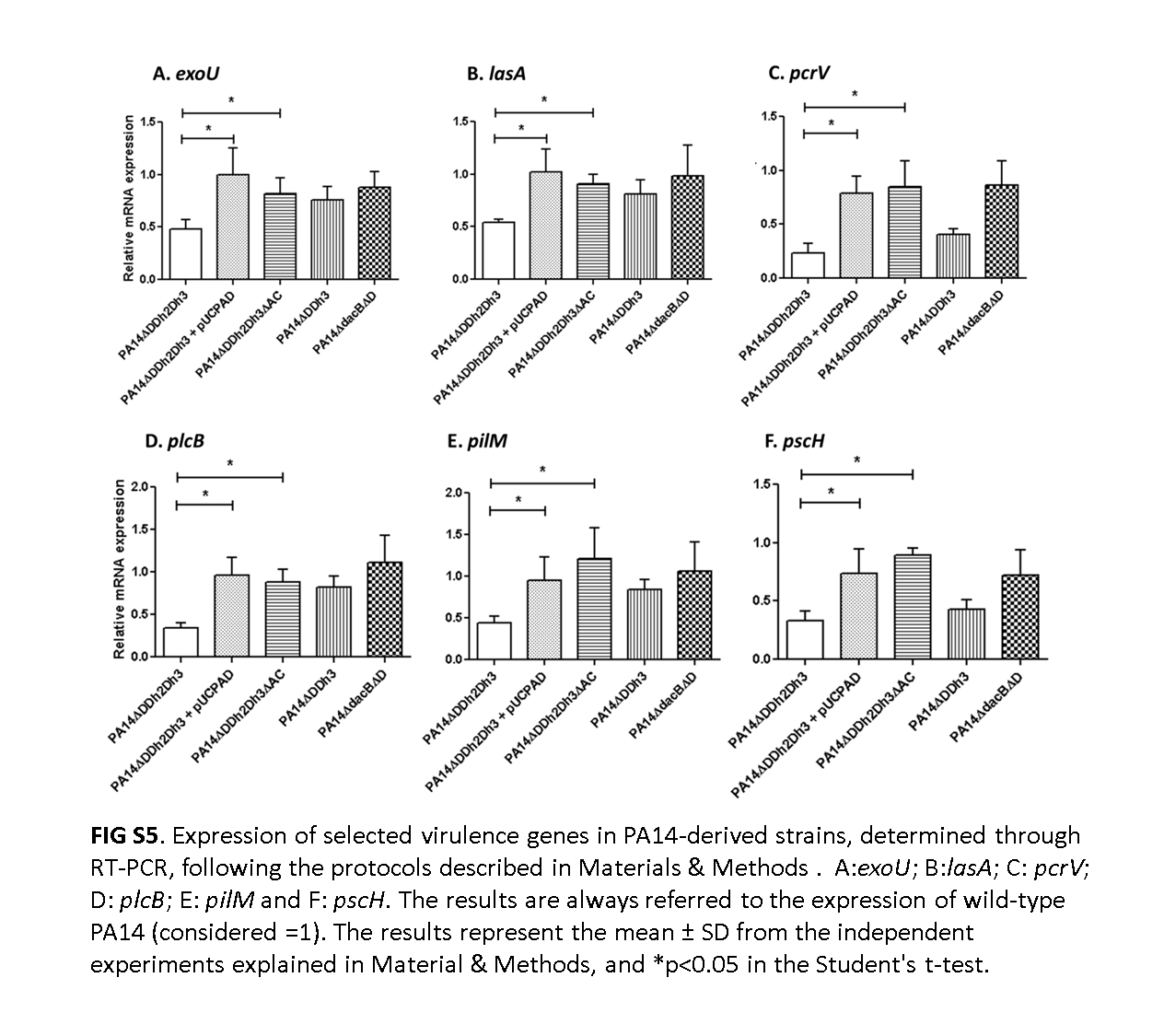

Supplement: Figure S5 — Expression of selected virulence genes in PA14-derived strains, determined through RT-PCR, following the protocols described in Materials and Methods. The virulence genes were exoU (A), lasA (B), pcrV (C), plcB(D), pilM (E), and pscH (F). The results are always relative to the expression of wild-type PA14 (set at 1). The results represent the means ± SD from the independent experiments conducted as described in Materials and Methods. *, P < 0.05 by Student’s t test. Download [file mbo005163047sf5.tif]

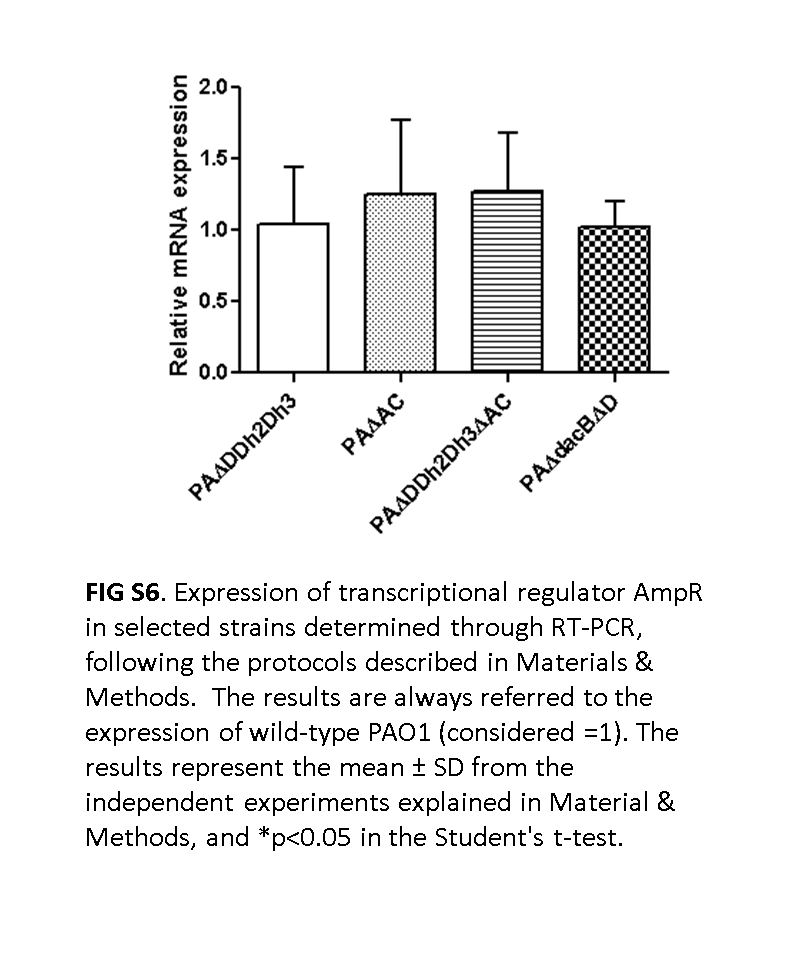

Supplement: Figure S6 — Expression of transcriptional regulator AmpR in selected strains determined through RT-PCR, following the protocols described in Materials and Methods. The results are always relative to the expression of wild-type PAO1 (set at 1). The results represent the means ± SD from the independent experiments conducted as described in Materials and Methods. *, P < 0.05 by Student’s t test. Download [file mbo005163047sf6.tif]
