# Supplementary material for: Impact of AmpC Derepression on Fitness and Virulence: the Mechanism or the Pathway?
Source: mBio. 2016 Oct 25;7(5):e01783-16. doi: 10.1128/mBio.01783-16 (PMC5080387; doi:10.1128/mBio.01783-16)
Supplement: Table S1 — Genes up- or downregulated in the triple ampD mutant compared to wild-type PAO1 strain. [file mbo005163047st1.docx]

**TABLE S1**. Genes up- or down-regulated in the triple *ampD* mutant compared to wild-type PAO1.

| **Gene number (PAO1**  **genome)** | **Fold increase/**  **decrease (PA∆DDh2Dh3 vs PAO1)^a^** | **Gene name** | **Description** | **Biologic function** |
| --- | --- | --- | --- | --- |
| **Up regulated** |  |  |  |  |
| PA0263 | 13 | *hcpC* | secreted protein Hcp | Secreted Factors (toxins, enzymes, alginate) |
| PA0456 | 3.15 |  |  | Probable cold-shock protein. Adaptation, protection; Transcriptional regulators |
| PA0525 | 2.63 |  |  | Probable dinitrification protein NorD. Energy metabolism |
| PA0730 | 2.74 |  |  | Probable transferase. Putative enzymes |
| PA0808 | 2.8 |  |  | Hypothetical, unclassified, unknown |
| PA0840 | 2.07 |  |  | Probable oxidoreductase. Putative enzymes |
| PA1183 | 3.85 | *dctA* | C4-dicarboxylate transport protein | Transport of small molecules |
| PA1323 | 3 |  |  | Hypothetical, unclassified, unknown |
| PA1324 | 4 |  |  | Hypothetical, unclassified, unknown |
| PA1471 | 2.12 |  |  | Hypothetical, unclassified, unknown |
| PA1562 | 2.3 | *acnA* | aconitate hydratase 1 | Energy metabolism |
| PA1592 | 2.67 |  |  | Hypothetical, unclassified, unknown |
| PA1746 | 2.5 |  |  | Hypothetical, unclassified, unknown |
| PA2007 | 3.58 | *maiA* | maleylacetoacetate isomerase | Carbon compound catabolism |
| PA2008 | 3.28 | *fahA* | fumarylacetoacetase | Carbon compound catabolism |
| PA2009 | 2.71 | *hmgA* | homogentisate 1,2-dioxygenase | Carbon compound catabolism |
| PA2562 | 2.43 |  |  | Hypothetical, unclassified, unknown |
| PA2788 | 2.05 |  |  | Probable chemotaxis transducer. Adaptation, protection; Chemotaxis |
| PA2840 | 4.05 |  |  | Probable ATP-dependent RNA helicase. Transcription, RNA processing and degradation |
| PA3031 | 2.38 |  |  | Hypothetical, unclassified, unknown |
| PA3392 | 2.03 | *nosZ* | nitrous-oxide reductase precursor | Energy metabolism |
| PA3393 | 2.6 | *nosD* | NosD protein | Energy metabolism |
| PA3394 | 2.78 | *nosF* | NosF protein | Energy metabolism; Transport of small molecules |
| PA3395 | 2.16 | *nosY* | NosY protein | Energy metabolism; Membrane proteins |
| PA3436 | 2.03 |  |  | Antibiotic biosynthesis monooxygenase |
| PA3460 | 2.31 |  |  | Probable acetyltransferase. Putative enzymes |
| PA3691 | 2.59 |  |  | Chromosome partitioning protein |
| PA3692 | 2.54 |  |  | Probable outer membrane protein. Membrane proteins |
| PA3819 | 3.26 |  |  | Hypothetical, unclassified, unknown; Membrane proteins |
| PA3888 | 2.12 |  |  | Probable permease of ABC transporter. Membrane proteins; Transport of small molecules |
| PA3890 | 2.55 |  |  | Probable permease of ABC transporter. Membrane proteins; Transport of small molecules |
| PA3891 | 2.02 |  |  | Probable permease of ABC transporter. Membrane proteins; Transport of small molecules |
| PA4110 | 123.22 | *ampC* | beta-lactamase precursor | Adaptation, protection |
| PA4111 | 21.48 |  |  | Hypothetical, unclassified, unknown |
| PA4141 | 2.02 |  |  | Hypothetical, unclassified, unknown |
| PA4154 | 2.21 |  |  | Hypothetical, unclassified, unknown |
| PA4292 | 2.02 |  |  | Probable phosphate transporter. Membrane proteins; Transport of small molecules |
| PA4394 | 2.1 |  |  | Hypothetical, unclassified, unknown |
| PA4433 | 2.01 | *rplM* | 50S ribosomal protein L13 | Translation, post-translational modification, degradation |
| PA4880 | 2.71 |  |  | Probable bacterioferritin. Central intermediary metabolism |
| PA4888 | 2.3 |  |  | Hypothetical, unclassified, unknown |
| PA5098 | 2.54 | *hutH* | histidine ammonia-lyase | Amino acid biosynthesis and metabolism |
| PA5100 | 2.42 | *hutU* | Urocanase | Amino acid biosynthesis and metabolism |
| PA5178 | 2.07 |  |  | Putative peptidoglycan-binding protein |
| PA5212 | 2.59 |  |  | Hypothetical, unclassified, unknown |
| PA5266 | 2.02 | *vgrG6* | type IV secretion protein | Protein secretion |
| PA5482 | 2.02 |  |  | Hypothetical, unclassified, unknown; Membrane proteins |
| **Down regulated** |  |  |  |  |
| PA0026 | -2.16 | *plcB* | phospholipase C, PlcB | Hypothetical, unclassified, unknown |
| PA0526 | -2.54 |  |  | Hypothetical, unclassified, unknown |
| PA0572 | -2.63 |  |  | Hypothetical, unclassified, unknown |
| PA0779 | -2.18 |  |  | Probable ATP-dependent protease. Putative enzymes |
| PA1070 | -2.31 | *braG* | branched-chain amino acid transport protein BraG | Transport of small molecules |
| PA1073 | -2.14 | *braD* | branched-chain amino acid transport protein BraD | Membrane proteins |
| PA1556 | -2.12 |  |  | Probable cytochrome c oxidase subunit. Energy metabolism |
| PA1596 | -3.34 | *htpG* | heat shock protein HtpG | Chaperones & heat shock proteins |
| PA1706 | -2.33 | *pcrV* | type III secretion protein PcrV | Protein secretion/export apparatus |
| PA1707 | -2.02 | *pcrH* | regulatory protein PcrH | Protein secretion/export apparatus; Secreted Factors (toxins, enzymes, alginate) |
| PA1708 | -2.22 | *popB* | translocator protein PopB | Protein secretion/export apparatus |
| PA1709 | -2.4 | *popD* | Translocator outer membrane protein PopD precursor | Protein secretion/export apparatus |
| PA1718 | -3.22 | *pscE* | type III export protein PscE | Protein secretion/export apparatus |
| PA1721 | -2.26 | *pscH* | type III export protein PscH | Protein secretion/export apparatus |
| PA1722 | -2.57 | *pscI* | type III export protein PscI | Protein secretion/export apparatus |
| PA1871 | -2.71 | *lasA* | LasA protease precursor | Translation, post-translational modification, degradation; Secreted Factors (toxins, enzymes, alginate) |
| PA3091 | -2.5 |  |  | Hypothetical, unclassified, unknown |
| PA3145 | -2.06 | *wbpL* | glycosyltransferase WbpL | Lipopolysaccharide biosynthesis. Cell wall / LPS / capsule; Membrane proteins |
| PA3149 | -2.75 | *wbpH* | probable glycosyltransferase WbpH | Lipopolysaccharide biosynthesis. Cell wall / LPS / capsule; Putative enzymes |
| PA3151 | -2.22 | *hisF2* | imidazoleglycerol-phosphate synthase, cyclase subunit | Histidine metabolism. Amino acid biosynthesis and metabolism |
| PA3152 | -2.08 | *hisH2* | glutamine amidotransferase | Histidine metabolism. Amino acid biosynthesis and metabolism |
| PA3327 | -2.13 |  |  | Probable non-ribosomal peptide synthetase. Adaptation, protection |
| PA3790 | -2.3 | *oprC* | Putative copper transport outer membrane porin OprC precursor | Transport of small molecules |
| PA4387 | -2.2 |  |  | Conserved hypothetical protein. Chaperones & heat shock proteins |
| PA4523 | -2.07 |  |  | Hypothetical, unclassified, unknown |
| PA4542 | -2.6 | *clpB* | ClpB protein | Translation, post-translational modification, degradation |
| PA4762 | -2.29 | *grpE* | heat shock protein GrpE | DNA replication, recombination, modification and repair; Chaperones & heat shock proteins |
| PA5039 | -2.19 | *aroK* | shikimate kinase | Amino acid biosynthesis and metabolism |
| PA5040 | -3.11 | *pilQ* | Type 4 fimbrial biogenesis outer membrane protein PilQ precursor | Motility & Attachment |
| PA5041 | -3.78 | *pilP* | type 4 fimbrial biogenesis protein PilP | Motility & Attachment |
| PA5042 | -4.03 | *pilO* | type 4 fimbrial biogenesis protein PilO | Motility & Attachment |
| PA5043 | -4.62 | *pilN* | type 4 fimbrial biogenesis protein PilN | Motility & Attachment |
| PA5044 | -3.27 | *pilM* | type 4 fimbrial biogenesis protein PilM | Motility & Attachment |
| PA5053 | -3.13 | *hslV* | heat shock protein HslV | Chaperones & heat shock proteins |

^a^ Genes with expression levels at least 2-fold higher/lower than those of wild-type PAO1
