# Supplementary material for: Impact of AmpC Derepression on Fitness and Virulence: the Mechanism or the Pathway?
Source: mBio. 2016 Oct 25;7(5):e01783-16. doi: 10.1128/mBio.01783-16 (PMC5080387; doi:10.1128/mBio.01783-16)
Supplement: Table S2 — Strains and plasmids used in this work. [file mbo005163047st2.docx]

**TABLE S2**. Strains and plasmids used in this work.

| **Strain or plasmid** | **Genotype/relevant characteristic(s)** | **Reference or source** |
| --- | --- | --- |
| ***P. aeruginosa*** |  |  |
| PAO1 | Completely sequenced reference strain | 1 |
| PAΔD | PAO1 ΔampD::*lox*; AmpD is an N-acetyl-anhydromuramyl–L-alanine amidase involved in peptidoglycan recycling; negative regulator of AmpC expression | 2 |
| PAΔDh2 | PAO1 ΔampDh2::*lox*; AmpDh2 is an additional AmpD homologue of *P. aeruginosa* | 2 |
| PAΔDh3 | PAO1 ΔampDh3::*lox*; AmpDh3 is an additional AmpD homologue of *P. aeruginosa* | 2 |
| PAΔDh2Dh3 | PAO1 ΔampDh2::*lox* ΔampDh3::lox | 2 |
| PAΔDDh2 | PAO1 ΔampD::*lox* ΔampDh2::*lox* | 2 |
| PAΔDDh3 | PAO1 ΔampD::*lox* ΔampDh3::*lox* | 2 |
| PAΔDDh2Dh3 | PAO1 ΔampD::*lox* ΔampDh2::*lox* ΔampDh3::*lox;* Mutant derepressed for AmpC production | 2 |
| PAΔDDh2Dh3∆AC | PAO1 ΔampD::*lox* ΔampDh2::*lox* ΔampDh3::lox ΔampC::*lox*; *ampC* encodes the chromosomal cephalosporinase of *P. aeruginosa*. | 3 |
| PA∆AC | PAO1 ΔampC::*lox* | 3 |
| PA∆dacB | PAO1 ΔdacB::*lox;* dacB encodes the nonessential penicillin-binding protein 4 | 4 |
| PAΔdacBΔD | PAO1 ΔdacB::*lox* ΔampD::*lox;* Mutant derepressed for AmpC production | 4 |
| PAΔAG | PAO1 ΔampG::*lox; ampG* encodes the specific permease allowing the entry of PGN derivatives into the cytosol of *P. aeruginosa*. | 5 |
| PAΔDDh2Dh3∆AG | PAO1 ΔampD::*lox* ΔampDh2::*lox* ΔampDh3::lox ΔampG::*lox* | This work |
| PAΔAR | PAO1 ΔampR::*lox; ampR* encodes the transcriptional regulator controlling AmpC expression of *P. aeruginosa*. | 4 |
| PAΔDDh2Dh3∆AR | PAO1 ΔampD::*lox* ΔampDh2::*lox* ΔampDh3::lox ΔampR::*lox* | This work |
| PA∆creBC | PAO1 *∆creBC::lox; creBC* encodes the two-component system global regulator CreBC (BlrAB) | 4 |
| PAΔdacBΔD∆creBC | PAO1 ΔdacB::*lox;* ΔampD::*lox;* *∆creBC::lox* | This work |
| **PA14** | Completely sequenced reference strain | 6 |
| PA14ΔD | PA14 ΔampD::lox | 7 |
| PA14ΔDh2 | PA14 ΔampDh2::lox | This work |
| PA14ΔDh3 | PA14 ΔampDh3::lox | This work |
| PA14ΔDh2Dh3 | PA14 ΔampDh2::lox ΔampDh3::lox | This work |
| PA14ΔDDh2 | PA14 ΔampD::lox ΔampDh2::lox | This work |
| PA14ΔDDh3 | PA14 ΔampD::lox ΔampDh3::lox | This work |
| PA14ΔDDh2Dh3 | PA14 ΔampD::lox ΔampDh2::lox ΔampDh3::lox; Mutant derepressed for AmpC production | This work |
| PA14ΔDDh2Dh3∆AC | PA14 ΔampD::lox ΔampDh2::loxΔampDh3::lox ΔampC::*lox* | This work |
| PA14∆AC | PA14 ΔampC::*lox* | This work |
| PA14∆dacB | PA14 ΔdacB::*lox* | 7 |
| PA14ΔdacBΔD | PA14 ΔdacB::*lox* ΔampD::*lox*  Mutant derepressed for AmpC production | This work |
| PA14ΔAG | PA14 ΔampG::*lox; ampG* encodes the specific permease allowing the entry of PGN derivatives into the cytosol of *P. aeruginosa*. | This work |
| PA14ΔDDh2Dh3∆AG | PA14 ΔampD::*lox* ΔampDh2::*lox* ΔampDh3::lox ΔampG::*lox* | This work |
| PA14ΔAR | PA14 ΔampR::*lox; ampR* encodes the transcriptional regulator controlling AmpC expression of *P. aeruginosa*. | This work |
| PA14ΔDDh2Dh3∆AR | PA14 ΔampD::*lox* ΔampDh2::*lox* ΔampDh3::lox ΔampR::*lox* | This work |
| **Plasmids** |  |  |
| pUCP24 | Gm^r^; pUC18-based *Escherichia-Pseudomonas* shuttle vector | 8 |
| pUCPAD | Gm^r^; pUCP24 containing PAO1 AmpD gene | 9 |
| pUCPAC | Gm^r^; pUCP24 containing PAO1 AmpC gene | 10 |
|  |  |  |

**TABLE S1 References**

1. Stover CK, Pham XQ, Erwin AL, Mizoguchi SD, Warrener P, Hickey MJ, Brinkman FS, Hufnagle WO, Kowalik DJ, Lagrou M, Garber RL, Goltry L, Tolentino E, Westbrock-Wadman S, Yuan Y, Brody LL, Coulter SN, Folger KR, Kas A, Larbig K, Lim R, Smith K, Spencer D, Wong GK, Wu Z, Paulsen IT, Reizer J, Saier MH, Hancock RE, Lory S, Olson MV. 2000. Complete genome sequence of *Pseudomonas aeruginosa* PAO1, an opportunistic pathogen. Nature 406:959–964. PMID: 10984043.
2. Juan C, Moyá B, Pérez JL, Oliver A. 2006. Stepwise upregulation of the *Pseudomonas aeruginosa* chromosomal cephalosporinase conferring high-level beta-lactam resistance involves three AmpD homologues. Antimicrob Agents Chemother 50:1780-1787. PMID: 16641450.
3. Moya B, Juan C, Albertí S, Pérez JL, Oliver A. 2008. Benefit of having multiple *ampD* genes for acquiring beta-lactam resistance without losing fitness and virulence in *Pseudomonas aeruginosa*. Antimicrob Agents Chemother 52: 3694-3700. PMID: 18644952.
4. Moya B, Dötsch A, Juan C, Blázquez J, Zamorano L, Haussler S, Oliver A. 2009. Beta-lactam resistance response triggered by inactivation of a nonessential penicillin-binding protein. PLoS Pathog 5: e1000353. PMID: 19325877.
5. Zamorano L, Reeve TM, Juan C, Moyá B, Cabot G, Vocadlo DJ, Mark BL, Oliver A .2011. AmpG inactivation restores susceptibility of pan-beta-lactam-resistant *Pseudomonas aeruginosa* clinical strains. Antimicrob Agents Chemother 55:1990-1996. PMID: 21357303.
6. Lee DG, Urbach JM, Wu G, Liberati NT, Feinbaum RL, Miyata S, Diggins LT, He J, Saucier M, Déziel E, Friedman L, Li L, Grills G, Montgomery K, Kucherlapati R, Rahme LG, Ausubel FM. 2006. Genomic analysis reveals that *Pseudomonas aeruginosa* virulence is combinatorial. Genome Biol 7: R90. PMID: 17038190.
7. Zamorano L, Moyá B, Juan C, Oliver A. 2010. Differential beta-lactam resistance response driven by *ampD* or *dacB* (PBP4) inactivation in genetically diverse *Pseudomonas aeruginosa* strains. J Antimicrob Chemother 65:1540-1542. PMID: 20435778.
8. Juan C, Maciá MD, Gutiérrez O, Vidal C, Pérez JL, Oliver A. 2005. Molecular mechanisms of beta-lactam resistance mediated by AmpC hyperproduction in *Pseudomonas aeruginosa* clinical strains. Antimicrob Agents Chemother 49:4733-4738. PMID: 16251318.
9. Juan C, Maciá MD, Gutiérrez O, Vidal C, Pérez JL, Oliver A. 2005. Molecular mechanisms of beta-lactam resistance mediated by AmpC hyperproduction in *Pseudomonas aeruginosa* clinical strains. Antimicrob Agents Chemother 49:4733-4738. PMID: 16251318.
10. Cabot G, Bruchmann S, Mulet X, Zamorano L, Moyà B, Juan C, Haussler S, Oliver A. 2014. *Pseudomonas aeruginosa* ceftolozane-tazobactam resistance development requires multiple mutations leading to overexpression and structural modification of AmpC. Antimicrob Agents Chemother 58: 3091-3099. PMID: 24637685.
