# Supplementary material for: Impact of AmpC Derepression on Fitness and Virulence: the Mechanism or the Pathway?
Source: mBio. 2016 Oct 25;7(5):e01783-16. doi: 10.1128/mBio.01783-16 (PMC5080387; doi:10.1128/mBio.01783-16)
Supplement: Table S3 — Primers used in this study. [file mbo005163047st3.docx]

**TABLE S3**. Primers used in this study.

| **Primer** | **Sequence (5′-3*′)***[**^a^**](http://aac.asm.org/content/50/5/1780/T2.expansion.html#fn-2) | **PCR product size (bp)** |
| --- | --- | --- |
| AC-rnaF | GGGCTGGCCTCGAAAGAGGAC | 246 |
| AC-rnaR | GCACCGAGTCGGGGAACTGCA |  |
| AR-rnaF | GCCACCAGGTGAAGAGCCTCG | 256 |
| AR-rnaR | ATGAAGGGATGGCGCGCCTGG |  |
| Exo-UrnaF | TTGGGGGCTACTGCCTCCTCG | 245 |
| Exo-UrnaR | AGAGTGATCTGCCGCGGCCCT |  |
| ExoS-rnaF | CGCCGTCGAATTGCACCAGGC | 251 |
| ExoS-rnaR | ACATGACCGCAGGCTGCGCGT |  |
| LasA-rnaF | GCTGTCGGCGAAACGCGGCTT | 250 |
| LasA-rnaR | GGTGCCGAACAGGCGCTGGAA |  |
| PcrV-rnaF | TTGCGCAGCGAGCGGATCGTG | 272 |
| PcrV-rnaR | CCGTCCTGGGTCTGCAGGACA |  |
| PlcB-rnaF | TGGCCCAGCCGCACCATGTGT | 250 |
| PlcB-rnaR | ATCAGCTCCTTCGCCACCCGC |  |
| PilM-rnaF | GCAGGGCGGTCTTCCGGATGA | 261 |
| PilM-rnaR | AGGGCGCCGGCATTCACCTTG |  |
| PscH-rnaF | ATGAGCCGCATCGACACGCCG | 259 |
| PscH-rnaR | GCGGCAGTTCCGCACGGAGCA |  |

^a^Sequences were obtained from the published PAO1 genome, or PA14 in the case of ExoU primers. For the rest of the genes, the sequences of amplicons were checked to be conserved in the two strains. The *ampC* and *rpsL* RT-PCR primers were described previously (1).

**TABLE S2 References**

1. Juan C, Moyá B, Pérez JL, Oliver A. 2006. Stepwise upregulation of the *Pseudomonas aeruginosa* chromosomal cephalosporinase conferring high-level beta-lactam resistance involves three AmpD homologues. Antimicrob Agents Chemother 50:1780-1787. PMID: 16641450.
